# Actomyosin rings constrain CD40 mobility to organize the dendritic cell immunological synapse

**DOI:** 10.1186/s12964-026-02816-0

**Published:** 2026-04-18

**Authors:** Camille D. Clamagirand, Eliane Mallet, Daniel J. Nieves, Christoph Ratswohl, Dylan M. Owen, Jérémie Rossy

**Affiliations:** 1 Institute of Cell Biology and Immunology Thurgau (BITG), University of Konstanz, Kreuzlingen, 8280 Switzerland; 2 Graduate School for Cellular and Biomedical Sciences, University of Bern, Bern, 3012 Switzerland; 3 Graduate School Health and Drugs Sciences, Université Paris-Saclay, Gif-Sur-Yvette, 91190 France; 4 Department of Immunology and Immunotherapy, School of Infection, Inflammation and Immunology, College of Medicine and Health, University of Birmingham, Birmingham, B15 2TT UK; 5 Centre of Membrane Proteins and Receptors, University of Birmingham, Birmingham, B15 2TT UK; 6 Department of Biology, University of Konstanz, Constance, 78457 Germany; 7 School of Mathematics, College of Engineering and Physical Sciences, University of Birmingham, Birmingham, UK

**Keywords:** Dendritic cell, CD40, Immunological synapse, Actin cytoskeleton, Licensing

## Abstract

**Background:**

The interaction between dendritic cell CD40 and T cell CD40L is critical for dendritic cell licensing and the initiation of adaptive immunity. The spatial organization of CD40 on the dendritic cell surface is thought to dictate its signaling output, yet the underlying mechanisms are poorly understood.

**Methods:**

Fluorescently tagged BMDCs were generated via lentiviral transduction of CD40-mGFP and electroporation of LifeAct-eGFP constructs. BMDCs, derived from C57BL/6 J mice and matured with LPS, were co-cultured with naïve OT-II CD4⁺ T cells or functionalized beads to model immunological synapses. Pseudo-synapse formation and CD40 recruitment were analyzed using TIRF, confocal, and dSTORM super-resolution microscopy, with actin dynamics and membrane protein mobility assessed via live-cell imaging and FRAP. Surface protein expression, cell viability, and cytokine secretion were quantified by flow cytometry and ELISA. Super-resolution data were analyzed using density-based clustering and Ripley’s L-function to quantify spatial organization. Statistical comparisons were performed using Student’s t-tests or one- and two-way ANOVA, with significance thresholds set at *p* ≤ 0.05.

**Results:**

We show that dendritic cell maturation first remodels the nanoscale clustering of CD40. Upon immunological synapse formation and CD40L engagement, CD40 is concentrated into a stable central cluster. We demonstrate that this centralization is not driven by global actin flow but by a specific restriction of CD40's lateral mobility at the synapse center. We identify contractile actomyosin rings, marked by α-actinin-4 and phosphorylated myosin light chain, encircling CD40. CD40 ligation increases myosin IIA activity, and pharmacological inhibition of this activity abrogates both the integrity of these rings and CD40 centralization. Functionally, disrupting this spatial organization selectively impairs the p38 MAPK pathway and the upregulation of costimulatory ligands CD70 and OX40L.

**Conclusions:**

Our findings reveal a myosin IIA-dependent mechanism that organizes CD40 to fine-tune dendritic cell licensing.

**Supplementary Information:**

The online version contains supplementary material available at 10.1186/s12964-026-02816-0.

## Background

Dendritic cells are critical for initiating adaptive immunity through T cell activation, a process orchestrated at a specialized interface known as the immunological synapse [7, 17, 22]. Within this junction, dendritic cells present antigenic peptides on major histocompatibility complex (MHC) molecules to the T cell receptor, providing the first signal for activation. However, T cell fate is ultimately determined by further instructions delivered by the dendritic cell in the form of co-stimulatory molecules and cytokines, which guide the nature and magnitude of the subsequent immune response [14, 56].

Signaling at the synapse is not unidirectional. T cells provide crucial feedback to dendritic cells, primarily through the interaction between members of the Tumor Necrosis Factor (TNF) superfamily [16]. The main example of this is the engagement of CD40 on the dendritic cell by its ligand, CD40L, expressed on activated CD4 + T helper cells. This interaction triggers a critical maturation program in the dendritic cell known as "licensing" [12, 35, 41]. Licensing, which is driven by the activation of NF-κB and mitogen-activated protein kinase (MAPK) pathways [62], upgrades the dendritic cell's capacity to prime cytotoxic CD8 + T cells, thereby linking the helper and cytotoxic branches of the adaptive immune response [45, 46].

The function of the immunological synapse is intimately linked to its highly organized molecular architecture. On the T cell side, the immunological synapse is characterized by a multi-scale organization where primary signaling units constituted of microclusters of receptors and adhesion molecules form upon antigen recognition and are subsequently transported to assemble the larger, classic 'bull's-eye' structure of the supramolecular activation cluster (SMAC) [18]. A similar principle of spatial organization governs the function of the TNFR superfamily, whose members must cluster to initiate a robust signal [28, 51]. CD40, for instance, does not exist as a simple monomer but is pre-organized into ligand-independent oligomers via a Pre-Ligand-Binding Assembly Domain (PLAD) [13]. Upon binding its trimeric ligand, these pre-formed units are driven into higher-order aggregates, a step that is essential for the efficient recruitment of downstream adaptors like TRAFs and the amplification of intracellular signaling [19, 52]. CD40 is further regulated by the upregulation of its expression during dendritic cell maturation [33], a crucial process that endows dendritic cells with the capacity to efficiently prime T cells. This increased receptor density not only enhances the signaling potential of the dendritic cell but may also alter the nanoscale patterning and clustering dynamics of CD40 at the plasma membrane, thereby influencing the threshold for T cell activation at the immunological synapse [33].

Dendritic cell maturation further involves a profound reorganization of the actin cytoskeleton, which is essential for stable synapse formation and effective T cell activation [3, 8, 30]. The actin cytoskeleton drives morphological changes at the immunological synapse and further restricts protein diffusion through a "picket fence" mechanism, where transmembrane proteins anchored to actin corrals create barriers to lateral movement [26, 49]. Furthermore, contractile actomyosin arcs, organized by non-muscle myosin II, can actively transport receptors, a mechanism shown to be critical for TCR centralization in T cells [59] and for organizing the B cell synapse [55]. Given that the actin-binding protein α-actinin can constrain the mobility of adhesion and signaling molecules [4, 15], it is plausible that the dendritic cell actomyosin network actively regulates the positioning of key receptors like CD40.

Here, we hypothesized that the spatial organization of CD40 within the dendritic cell immunological synapse is actively regulated and that this organization is essential for optimal signaling and dendritic cell licensing. Using the BMDC model on functionalized cover glass to gain mechanistic insight into the processes driving receptor organization at the dendritic cell synapse, we show that CD40L engagement drives the concentration of CD40 into a central cluster, a process mediated by a restriction of its lateral mobility. We identify contractile actomyosin rings, enriched in α-actinin-4 and active myosin IIA, that encircle the central CD40 cluster. Disrupting myosin IIA activity abrogates CD40 centralization and selectively impairs the p38 MAPK pathway and the upregulation of key costimulatory ligands. Our findings reveal a myosin IIA-dependent mechanism that spatially organizes CD40 to fine-tune dendritic cell licensing.

## Material and methods

### Plasmids

The plasmid pEGFP-C1-Lifeact-EGFP was a generous gift from Vladimir Purvanov, first published by Belin and colleagues [6] (Addgene, #58,470). The pLenti-CD40-mGFP was purchased from Origene (MR227116L4). The plasmids encoding for the VSVg pantropic glycoprotein pMD2.G and the psPAX2 were a generous gift from Richard Schreggle, obtained from Didier Trono (Addgene, #12,259,Addgene, #12,260).

### Generation of pseudo viral particles

Third-generation self-inactivating lentiviral particles were produced by transiently transfecting 293 T cells with a mixture of the lentiviral packaging plasmid psPAX2 (Addgene, #12,260), the envelope plasmid pMD2.G (Addgene, #12,259), and the desired construct pLenti-CD40-mGFP (Origen, MR227116L4) using the TransIT-LT1 transfection reagent (Mirus, MIR-2306). After 24 and 48 h of culture at 37 °C with 5% CO₂, the conditioned media from transfected 293 T cells were collected, and viral particles were precipitated overnight using a viral precipitation solution (8.5% PEG6000, 0.28 M NaCl, H₂O). The media were then centrifuged at 4400 g for 40 min at 4 °C, and the pellets were resuspended in PBS (Fisher BioReagents, BP399-20). Aliquots of the concentrated viral particles were stored at −80 °C until further use. The particles were titrated by transducing 293 T cells with serial dilutions of the aliquots. After 72 h of incubation at 37 °C with 5% CO₂, the percentage of GFP-positive 293 T cells was measured by flow cytometry, and the titer was calculated using Suwanai formula [25].

$$\mathrm{Titer}\left(\mathrm{IU/mL}\right)\;=\frac{\left(\#\;\mathrm{cells}\;\mathrm{at}\;\mathrm{starting}\;\mathrm{time}\right)\times\left(\mathrm{dilution}\;\mathrm{factor}\right)\times\left(\%\;\mathrm{GFP+}\;\mathrm{cells}\right)}{\left(\mathrm{volume}\;\mathrm{virus}\;\mathrm{solution}\;\mathrm{added}\;\mathrm{expressed}\;\mathrm{in}\;\mathrm{ml}\right)}$$

### Cell culture

#### Mice

The C57BL/6 J and OT-2/C57BL/6 J TCR transgenic mouse strains were bred and maintained at the University of Konstanz. For all experiments, both male and female mice aged between 8 and 14 weeks were utilized. Approval for organ collection was granted by the German Veterinary Authority, while animal experiments were conducted in accordance with the German Animal Protection Law, with authorization from the Review Board of the Regierungspräsidium Freiburg (T-21/03TFA and T-24/02TFA).

#### BMDCs

Bone marrow-derived dendritic cells (BMDCs) were generated from wild-type C57BL/6 J mice using an established method [32]. To obtain bone marrow cells, femurs and tibiae were flushed with phosphate-buffered saline (PBS) using a syringe. The collected cells were then centrifuged at 300 × g for 5 min. To remove red blood cells (RBCs), the pellet was treated with 1 × RBC lysis buffer (Biolegend, 420,301) for 30 s at room temperature (RT). The cell suspension was filtered through a 70 μm strainer to eliminate bone fragments and other debris, followed by neutralization with PBS. After another round of centrifugation, the cells were resuspended in R10 culture medium, which included RPMI 1640 (Pan Biotech, PANP04-18500), 2 mM L-glutamine (Pan Biotech, PANP04-82100), 100 U/mL penicillin, 100 μg/mL streptomycin (Pan Biotech, PANP06-07100), 10% heat-inactivated fetal calf serum (iFCS, Gibco, 10,270–106), and 50 μM β-mercaptoethanol (Gibco, 31,350–010). The cells were then plated at a density of 4 × 10⁶ cells/mL in 10 cm bacteriological Petri dishes containing 10 mL of R10 medium supplemented with 20 ng/mL murine granulocyte–macrophage colony-stimulating factor (GM-CSF,Peprotech, 315–03). On the third day of incubation at 37 °C with 5% CO₂ in a humidified environment, an additional 10 mL of R10 medium containing 20 ng/mL GM-CSF was added. After 3 more days, half of the culture medium was gently removed from the top and replaced with fresh R10 medium containing 20 ng/mL GM-CSF. By days 8 and 9 of differentiation, cells were classified as immature BMDCs. To promote their maturation, the cells were resuspended in R10 medium supplemented with GM-CSF and 100 ng/mL lipopolysaccharide (LPS) derived from Escherichia coli O111:B4 (Sigma-Aldrich, L4391) and incubated for 20 h. The purity and phenotype of the day 9 matured BMDC cultures were routinely confirmed by flow cytometry. The resulting population was homogeneously positive for CD11c and MHCII (CD11c +/MHCII-high), consistent with a pure population (> 90%) of conventional dendritic cells (Sup Fig. 1). To further confirm the classical dendritic cell identity and distinguish them from macrophages, we validated our protocol by assessing Zbtb46 expression. Our BMDC cultures consistently expressed significantly higher levels of the DC-specific transcription factor Zbtb46 compared to parallel bone marrow-derived macrophage cultures, unequivocally confirming their identity as classical dendritic cells (data to be published). For the experiment, one independent preparation of BMDCs was used for one repeat of experiment (n).

#### BMDCs-CD40-mGFP

To obtain BMDCs-CD40-mGFP, BMDCs were transduced on day 2 of the differentiation with pLenti-CD40-GFP using pseudoviral particles. Pseudoviral particles were added directly to the BMDC cultures at a multiplicity of infection (MOI) of 150, along with 100 µg/mL protamine sulfate (MP Biomedicals, 102,752). The rest of the differentiation process remained as previously described. Once matured with LPS, cells were washed 3 times in cold staining buffer (PBS containing 2% iFCS and 2 mM EDTA, AppliChem, A3145 0500) and cell number was adjusted to 2.5.10^6^ cells/mL, cells were then sorted for GFP-positive cells on a BD Aria IIu cell sorter. Immediately after sorting cells were resuspended in R10 culture medium and incubated at 37 °C with 5% CO₂. The cells were used for experiments on the same day.

#### BMDCs-LifeAct-eGFP

BMDCs were transfected using the Neon NxT Electroporation System (ThermoFisher). Electroporation was performed on day 9 of differentiation with three pulses at 1500 V for 10 ms, after resuspending the cells at 5 × 10⁶ cells/100 µL in Neon NxT Electrolytic E100 Buffer, together with 5 µg/100 µL of the LifeAct-eGFP plasmid. Immediately after electroporation, the cells were incubated for 6 h in R10 culture medium, supplemented with 20 ng/mL GM-CSF and 20% iFCS at a concentration of 2 × 10⁶ cells/mL. Following incubation, the cells were cultured in R10 medium with 20 ng/mL GM-CSF and 100 ng/mL LPS and incubated for 20 h.

#### T cells

Naïve OT-II CD4 + T cells were obtained from the spleens of OT-2/C57BL/6 J TCR transgenic mice. The spleens were first mechanically dissociated in PBS and passed through a 70 µm filter. The cells were then centrifuged at 300 × g for 5 min and the pellet was incubated in 1 × RBC lysis buffer for 30 s at room temperature (RT) and shortly washed with PBS. The resulting cell suspension was centrifuged at 300 × g for 10 min and then resuspended in staining buffer (PBS containing 2% iFCS and 2 mM EDTA (PanReac Aplichem, A3145-0500). T cells were further purified using the Mouse Naïve CD4⁺ T Cell Isolation Kit (Miltenyi Biotec, 130–104–453), following the manufacturer’s protocol. After isolation, the cells were cultured in R10 medium (RPMI 1640 with 2 mM L-glutamine, 100 U/mL penicillin, 100 μg/mL streptomycin, 10% heat-inactivated fetal calf serum (iFCS), and 50 μM 2-mercaptoethanol) supplemented with 0.4 U IL-7 (Peprotech, 217–17) and incubated at 37 °C with 5% CO₂. The cells were used for experiments the next day.

### BMDCs conjugation with coated beads

Beads (Polysciences, 17,136) were washed 3 times in borate buffer (0.1 M, pH 8.5) and centrifuged at 4000 g for 3 min before being resuspended in borate buffer with 400 µg/mL Alexa Fluor647 labeled anti-rat antibody (Invitrogen, A21247) per 2.25 × 10⁷ beads. The beads were then incubated overnight at room temperature (RT) with gentle end-to-end mixing. The following day, the beads were centrifuged for 5 min at 4000 g and resuspended in 10 mg/mL bovine serum albumin (BSA, Roth, 3737.1) diluted in borate buffer, then incubated for 1 h at RT with gentle mixing. The beads were centrifuged again at 4000 g for 3 min and resuspended in storage buffer (PBS with 10 mg/mL BSA, 0.1% NaN₃, 5% glycerol). Coated beads were stored at 4 °C until use. For the pseudo-synapse experiments, the coated beads were carefully washed twice with PBS and resuspended at 0.5 × 10⁶ beads/mL in PBS. Subsequently, 10 µg/mL of anti-MHCII (Invitrogen, 16–5321-85), anti-CD40 (Invitrogen, 16–0401-82), or their isotype control (Rat IgG2b kappa, Invitrogen, 16–4031-85) was added to the bead suspension. Meanwhile, BMDCs previously stained with CellTracker™ Blue CMHC (Invitrogen, C2111, 1:100) were resuspended in R10 culture media with 10 µM HEPES (HEPES (4-(2-hydroxyethyl)−1-piperazineethanesulfonic acid; Sigma-Aldrich, H0887). at a concentration of 1 × 10⁶ cells/mL. The cells were then added to the beads and incubated for 5 to 35 min at 37 °C. The number of BMDC-bead conjugates (BV421 + Alexa Fluor 647 +) from total beads (Alexa Fluor 647 +) was quantified using flow cytometry.

### Activation of BMDCs using coated beads

Beads (Polysciences, 17,136) were washed three times in borate buffer (0.1 M, pH 8.5) and centrifuged at 4000 × g for 3 min before being resuspended in either borate buffer supplemented with 0.01% poly-L-lysine (PLL; Sigma, P8920) to serve as control, or in borate buffer supplemented with 400 µg/mL anti-rat antibody (Invitrogen, 31,220) per 2.25 × 10^7^ beads. The beads were incubated overnight at RT with gentle end-to-end mixing. The following day, the beads were centrifuged for 5 min at 4000 × g and resuspended in 10 mg/mL bovine serum albumin (BSA; Roth, 3737.1) diluted in borate buffer, then incubated for 1 h at RT with gentle mixing. The beads were centrifuged again at 4000 × g for 3 min and resuspended in PBS at a final concentration of 1 × 10^7^ beads/mL. Subsequently, 10 µg/mL of anti-MHCII antibody (Invitrogen, 16–5321-85) or anti-ICAM1 (Invitrogen, 16–0542-81) was added to the anti-rat-coated bead suspension.

BMDCs, activated overnight with LPS and prepared from three mice were pooled and added to the prepared beads at a 1:1 bead-to-cell ratio. After 5 min of incubation at 37 °C, activation was stopped.

For the kinome activation assay, activation was stopped by adding ice-cold PBS, and the samples were briefly placed on ice. Samples were centrifuged for 5 min at 300 × g at 4 °C and washed once with ice-cold PBS. The resulting cell pellets were snap-frozen in liquid nitrogen and stored at − 80 °C prior to shipment to PamGene (PamGene International B.V., 's-Hertogenbosch, The Netherlands) for kinase activity analysis. Kinase profiling was performed using the PamGene® platform with PamChip® peptide microarrays operated on the PamStation® system. Briefly, samples were applied to arrays containing peptide substrates representing 196 protein tyrosine kinases (PTKs) or 144 serine/threonine kinases (STKs). Kinase-mediated phosphorylation of peptides was detected in real time using fluorescently labeled antibodies, and signal quantification was performed with BioNavigator™ software. Results are presented as a Kinase Coral Tree comparing kinase activity between anti-MHCII-coated and PLL-coated beads.

For the quantification of the phosphotyrosines, the activation was quickly stopped by adding pre-warmed gel sample buffer (225 mM Tris–HCl pH 6.8, 50% glycerol, 5% sodium dodecyl sulfate, 4% 2-mercaptoethanol, and 0.05% bromophenol blue) freshly supplemented with PhosphoSTOP (Roche, 04 906 837 001). The samples were then heated to 95 °C for 10 min and stored at −20 °C until use.

Protein separation was performed using 10% SDS-PAGE bis-acrylamide, followed by transfer onto PROTAN nitrocellulose membrane (GE Healthcare, 10,600,002) at 120 V for 1 h and 15 min. The membrane was briefly stained with Ponceau solution (5% acetic acid v/v, 0.1% Ponceau red w/v) to confirm proper protein transfer and to facilitate membrane cutting. After extensive washes with PBS-T (PBS + 0.05% Tween-20), membranes were blocked for 1 h with ROTI-Block solution (Carl Roth, A151.2). Membranes were then incubated overnight at 4 °C with anti-phosphotyrosines (pTyr, PY20, Invitrogen, 03–7700, 1:1000), the following primary antibodies, diluted in PBS-T containing 3% BSA and 0.02% NaN₃: The following day, membranes were washed 3 times in PBS-T, then incubated with HRP-conjugated anti-rabbit secondary antibody (Jackson ImmunoResearch, 111–035-003, 1:5000) diluted in PBS-T containing 5% milk powder. After 3 washes in PBS-T, membranes were developed using Clarity Western ECL Substrate (Bio-Rad, 170–5061).

For loading control acquisition, membranes were stripped with 0.2 M NaOH for 2 min, washed four times in PBS-T, and incubated with HRP-conjugated anti-β-actin antibody (Santa Cruz, sc-47778, 1:3000) diluted in PBS-T containing 5% milk powder for 1 h at room temperature. After 3 washes in PBS-T, membranes were developed again using Clarity Western ECL Substrate (Bio-Rad, 170–5061). Electrochemiluminescence signals were acquired using a ChemiDOC MP Imaging System (Bio-Rad), and quantification was performed using ImageLab (Bio-Rad) and Fiji ([44], v1.54p), with values normalized to β-actin as the loading control.

### Cytoskeleton inhibitors

LPS-matured BMDCs were resuspended at 1 × 10⁶ cells/mL in warm R10 culture medium supplemented with 10 µM HEPES. When indicated, the medium was further supplemented with either 50 µM Blebbistatin (Enzo Life Sciences, BML-EI315-0005), 100 µM CK666 (Abcam, Ab141231), 200 nM Cytochalasin D (Sigma, C8273) or dimethyl sulfoxide (DMSO) (Roth, A994.1).

### Microscopy

#### Pseudo-synapse model on glass

Glass coverslips (18 mm, #1.5, Marinefeld) were functionalized by coating them with 100 µg/mL human fibronectin (self-isolated from human sera) and 1 µg/mL CCL21 recombinant protein (Peprotech, 300–35), 10 µg/mL anti-MHCII antibody (Invitrogen, 16–5321-85),), 10 µg/mL anti-ICAM-1 antibody (eBioscience, 16.0542.81) and 10 µg/mL recombinant CD40L protein (R&D Systems, 8230-CL-050/CF), CD27 (R&D Systems, 574-CD-050), OX40 (R&D Systems, 1256-OX-050), 41BB (R&D Systems, 937-4B-050), LIGHT (R&D Systems, 1794-LT-025/CF) in PBS. The protein concentrations used for coating were empirically determined to be optimal for inducing robust synapse formation and were used consistently throughout the study. The coated coverslips were incubated at 37 °C for 30 min to 1 h, then washed 3 times with PBS. BMDCs were seeded at a density of 100 000 cells per coverslip in 1 mL of R10 culture medium and allowed to adhere and interact with the functionalized surface for 30 min at 37 °C in a 5% CO₂ atmosphere. Following incubation, the cells were fixed with 4% formaldehyde (Polysciences Inc., 18,814–20) at 37 °C for 20 min and later on stained for microscopy imaging. Pseudo-synapses were imaged using Total internal reflection fluorescence (TIRF) microscopy performed with a Leica DMi8 microscope fitted with a 100 ×/1.47 NA Plan-Apochromat oil immersion objective (Leica Microsystems, Wetzlar, Germany). Fluorescence detection was conducted with a DFC9000GTC sCMOS camera (Leica Microsystems). The imaging parameters and laser settings were managed through LAS X v3 software (Leica Microsystems).

#### Immunostaining for microscopy

Fixed cells were washed with PBS and permeabilized using 100 µg/mL Lysolecithin (Sigma Aldrich, L5254) for 10 min at room temperature (RT), followed by two additional PBS washes (5 min each). To reduce non-specific binding, the samples were blocked for 1 h at RT with PBS containing 5% bovine serum albumin (BSA, Roth, 3737.1). The cells were incubated overnight at 4 °C in the dark with antibodies diluted in the blocking solution from the following list: anti-CD40 (Abcam, ab212058, 1:100), anti-tubulin (Cytoskeleton, ATN02-S, 1:150), anti-actinin-4 (Invitrogen, 42–1400, 1:100), anti-phosphomyosin light chain (Cell Signaling Technology, 3671, 1:50), anti-CD70 (Abcam, ab223292, 1:100), anti-TRAF2 (Novus Biologicals, NBP1-86,913, 1:50) and anti-ICAM-1 (Invitrogen, MA5405, 1:50). The following day, the samples were washed 3 times with PBS-T (PBS with 0.05% Tween-20 (5 min per wash) (Sigma, P1379) and subsequently incubated for 1 h at RT in the dark with fluorescently labeled secondary antibodies and dyes from the following list: anti-rabbit-Alexa Fluor 488 (Jackson ImmunoResearch, 111.546.046, 1:250), anti-sheep-Alexa Fluor 6 47 (Invitrogen, A21448, 1:100), phalloidin-647 (Invitrogen, A22287, 1:250), anti-hamster-Alexa Fluor 647 (Jackson ImmunoResearch, 127–605-160, 1:100) and anti-rabbit-Alexa Fluor 647 (Invitrogen, A21246, 1:100). Following 3 final washes with PBS (5 min each), the samples were either imaged using TIRF microscopy on the same day or mounted on a slide with ProLong Diamond mounting media (Invitrogen, P36965) and stored at 4 °C in the dark until imaging with confocal microscopy.

#### dSTORM

##### dSTORM acquisition

BMDCs were seeded onto functionalized µ-Slide 18-well glass bottom (Ibidi, 81,817) and stained for CD40, as previously described. Shortly before imaging, the dSTORM imaging buffer (ONI) was reconstituted according to the manufacturer’s instructions and added to the well to be imaged. Acquisition began immediately of 256 × 256 images with a pixel size of 80 nm using a Deltavision OMXv4 Blaze using an Olympus 60x/1.49 UIS2 APON TIRFM objective and a pco sCMOS Camera. Fluorophore conversion was achieved using a 642 nm laser (100%). A 405 nm laser was used intermittently (10–100%) to reactivate fluorophores and sustain stochastic blinking throughout the acquisition.

##### dSTORM data processing

Localization was performed using SoftWoRx 7.0 software. Point spread function (PSF) width was set to 1.55 pixels, with an overlap threshold of 0.15. Localization event detection was carried out using maximum likelihood estimation (MLE). Drift correction was applied post hoc using absolute image correlation, with a pixel size of 10 nm and a time window interval of 200 s. After localization and drift correction, single-molecule tracks were assigned, and localizations were filtered based on localization precision and track length (1 to 5 frames). Final image reconstruction was performed by rendering each localization as a Gaussian function on a 10 nm lattice.

Single-molecule localization data were analyzed using an automated clustering pipeline implemented in R. The analysis utilized the packages; 'dbscan' for density-based clustering, 'spatstat' for spatial point pattern analysis, and 'raster' and 'sf' for spatial data handling. Data were first imported from CSV format and visualized using 2D histogram plots generated by the 'hist2d' function with a logarithmic intensity scale. Regions of interest (ROIs) were manually selected, and to facilitate localized clustering, a grid-based ROI segmentation approach was employed. The large manually defined ROI was divided into smaller subregions of 3000 × 3000 nm. The spatial polygons corresponding to the subregions were identified and filtered to retain only those entirely within the initial manually selected ROI. The single-molecule localizations within each subregion were extracted and saved as individual CSV files for subsequent analysis.

##### dSTORM analysis

Each segmented ROI was analyzed independently using the DBSCAN (Density-Based Spatial Clustering of Applications with Noise) algorithm [20], implemented through the 'dbscan' function. The clustering parameters were set to an epsilon (eps) value of 20 nm and a minimum points (minPts) threshold of 5, ensuring the identification of dense molecular clusters while filtering out noise. The clustering results were visualized by plotting individual localizations and overlaying convex hulls around identified clusters. Convex hull areas were computed for each cluster using the 'chull' function and the 'Polygon' function from the 'sf' package. To assess spatial clustering patterns at a global level, Ripley's L-function was calculated using the 'Lest' function from 'spatstat' with a maximum radius of 2200 nm. The deviation of L(r) from r was determined, and the maximum deviation value was extracted as a quantitative measure of clustering strength. Additionally, cluster density metrics, including the number of clusters per unit area and the number of localizations per cluster, were computed. Histograms summarizing these distributions were generated and saved alongside CSV files containing numerical results. Final outputs included per-cluster statistics such as localization density, cluster area, and estimated cluster radii.

#### CD40 recruitment in BMDCs-T cells conjugates

Glass coverslips were coated with 0.01% (v/v) Poly-L-Lysine (PLL, Sigma-Aldrich, P4832) for at least 30 min at 37 °C. The coverslips were then washed twice with PBS. Meanwhile, LPS-matured BMDCs were incubated for 1 h at 37 °C with 1 µM OVA 323–339 (Sigma O1641) at a concentration of 0.5 × 10⁶ cells/mL in R10 culture medium supplemented with 10 µM HEPES. After extensive washes in warm media, BMDCs and naïve OTII CD4 + T cells were placed in co-culture at a 1:1 ratio in R10 culture medium containing 10 µM HEPES. Cells were centrifuged for 30 s at 1000 g, then quickly placed at 37 °C for 30 min to allow conjugates formation. After incubation, the cells were carefully resuspended and seeded onto the washed PLL-coated coverslips. To ensure proper adhesion, the seeded cells were centrifuged at 90 g for 2 min. Conjugates were fixed with 4% formaldehyde (Polysciences Inc., 18,814–20) for 20 min at 37 °C and subsequently stained for microscopy. The recruitment of CD40 to the synapse was quantified using a Nikon Eclipse Ti2-E confocal microscope equipped with a 60X Plan Apo LambdaD immersion oil objective, using 488 nm and 640 nm excitation lasers.

#### Live-imaging of BMDCs

Glass coverslips were functionalized with 10 µg/mL anti-MHCII antibody (Invitrogen, 16–5321-85) and 10 µg/mL recombinant CD40L protein (R&D Systems, 8230-CL) as described previously. After washing, the cover glasses were placed in the microscope. Meanwhile, BMDCs were centrifuged at 300 × g for 5 min and resuspended in warm R10 culture medium supplemented with 10 µM HEPES. Just before starting the image acquisition, 50 000 BMDCs were added onto the glass. Images were captured every 30 s for BMDCs-CD40mGFP and every second for BMDCs-LifeAct-eGFP. Atmosphere was controlled and maintained at 37 °C in a 5% CO₂ atmosphere by using a live-cell incubation chamber (Pecon).

#### Fluorescence recovery after photobleaching (FRAP)

Cover glasses and BMDCs-CD40-mGFP were prepared as described for live imaging. For plasma membrane quantification, cells were stained with Cell Mask Deep Red Plasma Membrane (ThermoFisher, C10046) for 5 min, washed and used directly for experiments. Bleaching was performed using the Leica DMi8 microscope's scanner. After initial acquisition of 2 min, a rectangular region covering the cell diameter was bleached using a 488 nm argon laser (100% transmission), and recovery was recorded every 20 s for 15 min. Fluorescence intensities were quantified for each time point in three different regions of interest (ROIs). One ROI was defined in the bleached center of the cell, one in the bleached periphery, and one in the non-bleached periphery to compensate for photobleaching caused by repetitive imaging. The fluorescence intensity of the non-bleached periphery was subtracted from the intensities of the other two ROIs. The initial fluorescence intensity after bleaching was set to “0” and subsequently subtracted from all the values of the other ROIs. Results were further normalized by setting the initial fluorescence intensity before photobleaching to “1”.

Recovered fluorescence after 15 min was considered the mobile fraction (MF) for each condition and used for the calculation of the restriction index (RI) as follows:

$$\mathrm{RI}=\mathrm{MF}\left(\mathrm{periphery}\right)/\mathrm{MF}\left(\mathrm{center}\right).$$

### TIRF image analysis

Images acquired using TIRF microscopy were analyzed with Fiji [44], v1.54p). Cell size was defined using the actin channel. For quantification of the mean fluorescence intensity (MFI), cell outlines were defined using the actin channel, and the MFI of the signal was measured in the channel of interest. When specified, the MFI was normalized to the control (MFI ratio).

To quantify the MFI distributions in cells, we first calculated the centroid of the cells and applied 50 steps from the centroid to the periphery, following the outlines of the cells. The area between two steps was then defined as one region of interest (ROI) and as a distance of 2 arbitrary units (AU). The MFI of each ROI in each cell was then determined and divided by the MFI of the respective inner ROI of each cell. The ROIs were then averaged across cells from the same experiment, and statistics were performed for 3 independent experiments.

Actin speed quantification was performed on live TIRF images acquired from BMDCs-LifeAct-eGFP. After unsharpening the images (radius: 5 nm, mask: 0.8), a kymograph was generated for each cell by drawing a line from the center to the periphery and applying the ‘KymographBuilder’ function in ImageJ. On the resulting kymographs, actin speed was measured based on visible fibers. Fibers originating in the first third of the radius were classified as central, while those in the last third were categorized as peripheral.

The number of cells containing ACTN4 sarcomeric patterns was manually counted by 3 independent counters in a blinded and randomized manner. The counts were averaged and statistically analyzed.

### Impact of Blebbistatin treatment

BMDCs were treated with Blebbistatin as previously described. Cells were then incubated for 1 h at 37 °C. Cells were either plated for subsequent immunostaining for microscopy or analyzed by flow cytometry using Sytox Green (Invitrogen, S7020, 1:10,000), anti-CD40-APC (Biolegend,145,612, 1:100) and anti-MHCII-PE (Biolegend, 107,607, 1:800) in cold staining buffer (PBS containing 2% iFCS and 2 mM EDTA, Sigma-Aldrich). The cells were incubated with the antibodies at 4 °C in the dark for 30 min, then washed thoroughly in cold staining buffer before flow cytometry analysis.

### Licensing in vitro experiments

#### Western blot

BMDCs were treated with Blebbistatin as previously described. Cells were then incubated for 1 h at 37 °C before being stimulated with 10 µg/mL anti-CD40 antibody (Invitrogen, 16–0401-82) for 5 min at 37 °C with shaking (300 rpm) or with 10 nM of CCL19 [5] for 2 min at 37 °C with shaking (300 rpm). The reaction was quickly stopped by adding pre-warmed gel sample buffer (225 mM Tris–HCl pH 6.8, 50% glycerol, 5% sodium dodecyl sulfate, 4% 2-mercaptoethanol, and 0.05% bromophenol blue) freshly supplemented with PhosphoSTOP (Roche, 04 906 837 001). The samples were then heated to 95 °C for 10 min and stored at −20 °C until use. Protein separation, immunoblotting and quantifications were performed as described previously using the following primary antibodies, diluted in PBS-T containing 3% BSA and 0.02% NaN₃: anti-phospho-ERK (Cell Signaling, 4376, 1:1000), anti-phospho-p38 (Cell Signaling, 9211, 1:1000), anti-phospho-NFκB (Cell Signaling, 3033, 1:1000), anti-phospho-JNK (Cell Signaling, 9251, 1:1000). The membranes were developed using Clarity Western ECL Substrate (Bio-Rad, 170–5061) or SuperSignal West Femto Maximum Sensitivity Substrate (Pierce, 34,096).

#### Flow cytometry

##### Surface staining

Surface expression of proteins was assessed by flow cytometry using an LSR II flow cytometer and the following antibodies: anti-MHCII-FITC (BioLegend, 107,605, 1:500), anti-CD11c-BV421 (BioLegend, 117,330, 1:200), anti-CD70-APC (BioLegend, 104,610, 1:100), and anti-OX40L-APC (BioLegend, 108,811, 1:100). Staining was performed as stated before.

##### Viability

LPS-matured BMDCs were resuspended at 1 × 10⁶ cells/mL in warm R10 culture medium supplemented with 10 µM HEPES. When indicated, the medium was further supplemented with either 50 µM Blebbistatin (Enzo Life Sciences, BML-EI315-0005) or its vehicle, dimethyl sulfoxide (DMSO) (Roth, A994.1), and stimulated with 10 µg/mL anti-CD40 antibody (Invitrogen, 16–0401-82). Cells were incubated for 6 h before washed and resuspended in staining buffer (PBS containing 2% iFCS and 2 mM EDTA, Sigma-Aldrich) with 1 µM Sytox Blue (Invitrogen, S34857) immediately, 24 h, or 48 h after licensing. Fluorescence intensity was immediately measured using flow cytometry. Data were analyzed using FlowJo V10 software (Tree Star).

#### ELISA

LPS-matured BMDCs were resuspended at a concentration of 1 × 10⁶ cells/mL in pre-warmed R10 culture medium containing 10 µM HEPES (4-(2-hydroxyethyl)−1-piperazineethanesulfonic acid; Sigma-Aldrich, BML-EI315-0005). The medium was supplemented according to the indicated treatment with either 50 µM Blebbistatin (Enzo Life Sciences, BML-EI315-0005) or an equivalent volume of its vehicle, dimethyl sulfoxide (DMSO) (Roth, A994.1), along with 10 µg/mL anti-CD40 antibody (Invitrogen, 16–0401-82). Cells were incubated for 6 h at 37 °C with 5% CO₂, then washed 3 times in R10 culture medium before being further incubated for 42 h at 37 °C with 5% CO₂. The culture supernatants were then harvested and used directly for ELISA, the cells were meanwhile tested for viability (see section below). The release of IL-12 was assessed using the Murine IL-12 Standard ABTS ELISA Development Kit (PeproTech, 900-K97) according to the manufacturer’s instructions, with the exception that detection was performed using Streptavidin–Horseradish Peroxidase (Strp-HRP, Invitrogen, 434,323, 1:2500). For this, Strp-HRP was incubated for 30 min at room temperature (RT). After multiple washes with PBS-T, TMB substrate was added and incubated for 15 min at RT in the dark, followed by rapid termination of the reaction with 1 M H₂SO₄. Absorbance was measured at 450 nm using a Spark Multimode Microplate Reader (Tecan). To quantify the signals, a standard curve was generated from serial dilutions, and sample concentrations were calculated accordingly.

### Statistical analysis

Statistical analyses and data visualization were conducted using Prism v10 software (GraphPad, San Diego, CA, USA). Group differences were assessed using either unpaired or paired Student’s t-tests, while one-way or two-way analysis of variance (ANOVA) was applied for comparisons involving more than two groups. A significance threshold of 5% was used, with significance levels defined as follows: not significant (ns) for *p* > 0.05, * for *p* ≤ 0.05, ** for *p* ≤ 0.01, *** for *p* ≤ 0.001, and **** for *p* ≤ 0.0001.

## Results

### CD40 receptor organization is modified during maturation of BMDCs, and its engagement impacts synapse architecture

**Fig. 1:**
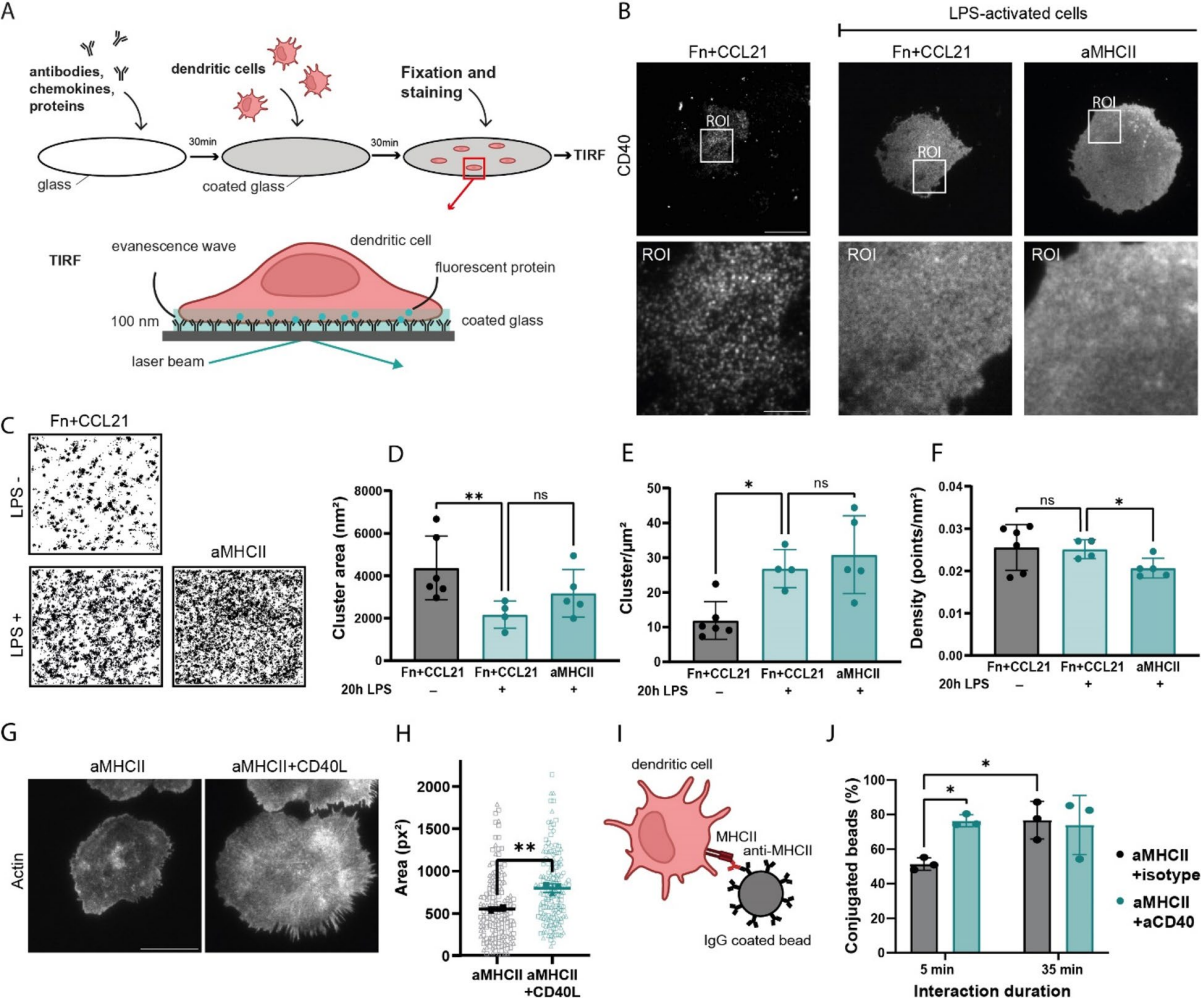
CD40 receptor organization is modified during maturation of BMDCs, and its engagement impacts synapse architecture. **A** Schematic of the pseudo-synapse model for TIRF microscopy. **B** Representative TIRF images of CD40 on LPS-matured BMDCs seeded on glass coated with either Fibronectin + CCL21 (Fn + CCL21) or anti-MHCII (aMHCII). White boxes indicate the regions of interest (ROI) used for subsequent dSTORM acquisition. Scale bar = 10 µm (*n* = 3). **C** Reconstructed dSTORM images showing CD40 localization within 3 µm × 3 µm ROIs from cells shown in (**B**). **D**-**F** Quantification of CD40 nanocluster parameters derived from dSTORM analysis: (**D**) cluster area, (**E**) cluster number per µm^2^, and (**F**) CD40 molecular density within clusters (each dot represents one cell, n = 3, unpaired Student’s t-test). **G** Representative TIRF images of F-actin (phalloidin stain) in BMDCs after 30 min on glass coated with aMHCII or aMHCII + CD40L. Scale bar = 20 µm. **H** Quantification of the BMDC spreading area from (**G**) (unpaired Student’s t-test, *n* = 3, n_1_ = 37 cells, n_2_ = 140 cells, n_3_ = 154 cells). **I** Schematic of the bead-cell conjugation assay. **J** Quantification of bead–cell conjugates after 5 or 35 min of interaction, with or without CD40 engagement (Two-way ANOVA, *n* = 3). Bars indicate mean ± SD. *p < 0.05, **p < 0.01; ns, not significant

While dendritic cell maturation is known to upregulate CD40 expression [33], how this translates to changes in the receptor's nanoscale spatial organization is unclear. To address this, we used a single-molecule approach: the direct stochastic optical reconstruction microscopy (dSTORM). Immature and LPS-matured BMDCs were seeded on functionalized glass surfaces reproducing two distinct contexts: glass coated with fibronectin and the chemokine CCL21 (Fn + CCL21) to model migratory conditions, and glass coated with functional anti-MHCII to trigger the formation of immunological synapses (Fig. 1A, B) [34]. This anti-MHCII stimulation induced robust synapse formation, evidenced by broad tyrosine's phosphorylation (Sup Fig. 2A, B), kinase activation (Sup Fig. 2C) and extensive cell spreading that was nearly four-fold greater than the adhesion-mediated spreading observed on surfaces coated with a functional antibody against ICAM-1 (Sup Fig. 2D, E) altogether confirming that anti-MHCII provides a potent signaling stimulus. We then performed cluster analysis of single-molecule localizations from dSTORM imaging of cells stained for CD40 (Fig. 1C, D, E, F).

Analysis of the Ripley’s K-function peak [39] indicated that CD40 molecules were significantly more clustered in immature than in mature cells (Sup Fig. 3A, B), where CD40 clusters were less numerous but larger (Fig. 1D, E). By contrast, synapse formation on anti-MHCII-coated glass did not significantly affect cluster size or number but did slightly reduce the molecular density within clusters (Fig. 1F). In parallel, we confirmed a higher expression of CD40 at the plasma membrane of mature BMDCs (Sup Fig. 3C, D). These data indicate that the nanoscale organization of CD40 at the plasma membrane of BMDCs is regulated primarily by maturation and to a lesser extent by synapse formation. They further suggest that the remodeling of CD40 clusters upon dendritic cell maturation could functionally reprogram the receptor, simultaneously raising the activation threshold for high-avidity T cell interactions and increasing its lateral mobility to facilitate recruitment to the site of T cell engagement.

Since we observed only a modest change in CD40 distribution upon synapse formation, we next examined synapses in which CD40 was actively engaged by its ligand, CD40L. We observed an increase in the synapse surface area of mature BMDCs (hereafter referred to as BMDCs) on anti-MHCII + CD40L-coated surfaces compared to those on anti-MHCII alone (Fig. 1G, H). This enlargement was specific to CD40 engagement, as synapses formed on surfaces with anti-MHCII and recombinant 41BB showed a reduction in surface area (Sup Fig. 3E, F). To understand the consequences of larger immunological synapses upon CD40 triggering, we incubated BMDCs with beads coated with either anti-MHCII and a functional antibody against CD40, or anti-MHCII and an isotype control, and measured the number of BMDC-bead conjugates by flow cytometry (Fig. 1I). Beads coated with anti-CD40 led to a higher number of conjugates at 5 min than those without CD40 engagement (Fig. 1J). This difference was not observed at 35 min, indicating that CD40 plays a role in the early stages of synapse formation. Altogether, our data show that BMDC maturation regulates the nanoscale organization of CD40 and that subsequent CD40L engagement modulates the formation of the immunological synapse.

### CD40L engagement drives CD40-specific centralization at the immunological synapse

**Fig. 2:**
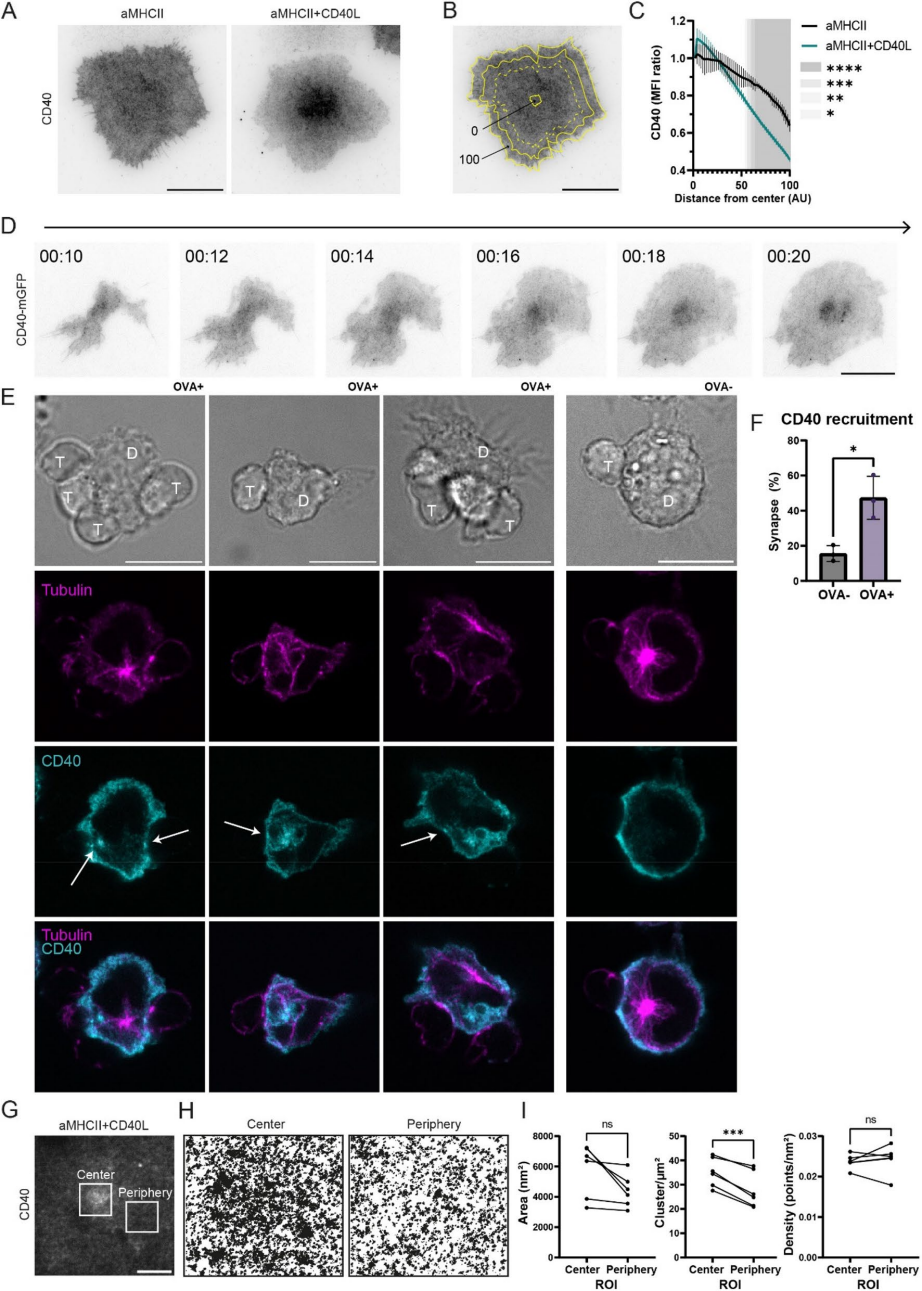
CD40 triggering promotes its concentration at the center of the synapse. **A** Representative TIRF images of CD40 on LPS-matured BMDCs after 30 min on glass coated with anti-MHCII (aMHCII) or aMHCII + CD40L. Scale bar = 20 µm. **B** Schematic illustrating the method used for radial profile quantification shown in (**C**). **C** Quantification of CD40 mean fluorescence intensity (MFI) as a function of normalized distance from the cell center (0 = center, 100 = periphery). Bars on the line represent SD from three independent experiments (Two-way ANOVA, n_1_ = 100 cells, n_2_ = 101 cells, n_3_ = 97 cells). **D** Representative time-lapse TIRF imaging of a CD40-mGFP-expressing BMDC landing on an aMHCII + CD40L-coated surface. Timestamp is hh:mm. Scale bar = 20 µm. **E** Representative confocal slices images of interactions between OT-II T cells (**T**) and BMDCs (**D**) either primed with OVA peptide (OVA +) or unprimed (OVA-). Cells are stained for tubulin (magenta) and CD40 (cyan). Arrows indicate CD40 accumulation at the synapse. Scale bar = 10 µm. **F** Quantification of the percentage of synapses showing CD40 recruitment from (**E**) (Unpaired Student’s t-test, n_1_ = 92 cells, n_2_ = 99 cells, n_3_ = 105 cells). **G** Representative TIRF image of a BMDC on aMHCII + CD40L showing the central and peripheral ROIs used for dSTORM imaging. Scale bar = 10 µm. **H** Reconstructed dSTORM images of CD40 distribution within the center and periphery ROIs shown in (**G**). **I** Quantification of CD40 nanocluster area, cluster density, and molecular density for central versus peripheral ROIs (each dot represents a single cell, paired t-test). In all graphs, bars indicate mean ± SD from three independent experiments (*n* = 3). *p < 0.05, **p < 0.01, ***p < 0.001, ****p < 0.0001; ns, not significant

Formation of the immunological synapse at the T cell side leads to a drastic reorganization of surface receptors, including nanoscale clustering and the formation of microscale supramolecular signaling clusters [17, 43]. The impact of CD40L on the dendritic cell synapse size therefore prompted us to investigate how this interaction affects the distribution of CD40 itself. TIRF imaging of BMDCs forming synapses on glass coated with anti-MHCII and CD40L revealed a distinct centralization of CD40 in the presence of its ligand (Fig. 2A). To better assess this distribution, we quantified the CD40 mean fluorescence intensity (MFI) in concentric areas from the cell center to the periphery (Fig. 2B, Sup Fig. 4A). This effect was specific to the CD40-CD40L interaction, as the presence of other TNF superfamily members, such as CD27, 41BB, OX40, or LIGHT, did not trigger CD40 concentration (Sup Fig. 4B, C, D). This density profile analysis confirmed that while CD40 was homogeneously distributed on anti-MHCII alone (from 1.00 ± 0.00 at the center to 0.64 ± 0.03 at the periphery), it became significantly concentrated in the center when CD40L was present (1.00 ± 0.00 at the center to 0.46 ± 0.01 at the periphery) (Fig. 2C). Consistent results were observed in experiments performed on anti-CD40-coated glass surfaces, further supporting the notion that CD40 redistributes and concentrates upon engagement (Sup Fig. 4E, F). This relocalization was not a general phenomenon, as the distribution of CD70, another TNF superfamily member, was unaffected by the presence of CD40L (Sup Fig. 4G, H). Recruitment of TRAF proteins, and especially TRAF2, to the CD40 cytoplasmic tail has been shown to be critical for downstream signaling [31, 54]. To assess whether this mechanism is engaged in our system, we examined TRAF2 localization at the immunological synapse. Consistent with its known role, we observed that TRAF2 is recruited and concentrated at the synapse upon CD40 engagement, indicating that CD40 signaling is likely initiated and properly organized at these sites (Sup Fig. 4I, J).

To understand the dynamics of this process, we performed live TIRF imaging of CD40-mGFP-expressing BMDCs on a surface coated with anti-MHCII and CD40L (Fig. 2D, Sup Movie 1). Centralization of CD40 began early during synapse formation (at approximately 10 min) and resulted in a stable central cluster by 20 min. We next validated these findings in dendritic-T cell conjugates by loading BMDCs with OVA peptide and incubating them with CD4^+^ OT-II transgenic T cells for 30 min. Confocal microscopy of these conjugates confirmed a punctate accumulation of CD40 at the center of the dendritic cell synapse, alongside a visible intracellular pool (Fig. 2E, Sup Movie 2). This recruitment was observed in a significantly higher number of synapses when BMDCs presented the OVA peptide compared to unloaded BMDCs (47.3% ± 4.5% vs. 15.6% ± 12.3%, respectively), confirming the process is linked to functional synapse formation (Fig. 2F).

Finally, we performed dSTORM microscopy to determine if this central accumulation was caused by a change in the nanoscale clustering of CD40. We compared central and peripheral regions of the synapse (Fig. 2G, H) and found that while cluster size and molecular density were similar in both areas, the number of clusters was significantly higher in the central region (Fig. 2I). This suggests that CD40L ligation does not alter the intrinsic nanoscale organization of CD40 but instead modulates the mobility of pre-existing clusters, confining them to the center of the synapse. In summary, our data show that the CD40-CD40L interaction actively reshapes CD40’s spatial organization during synapse formation, resulting in its specific concentration at the synapse center.

### Ligand engagement restricts CD40 mobility at the center of the immunological synapse

**Fig. 3:**
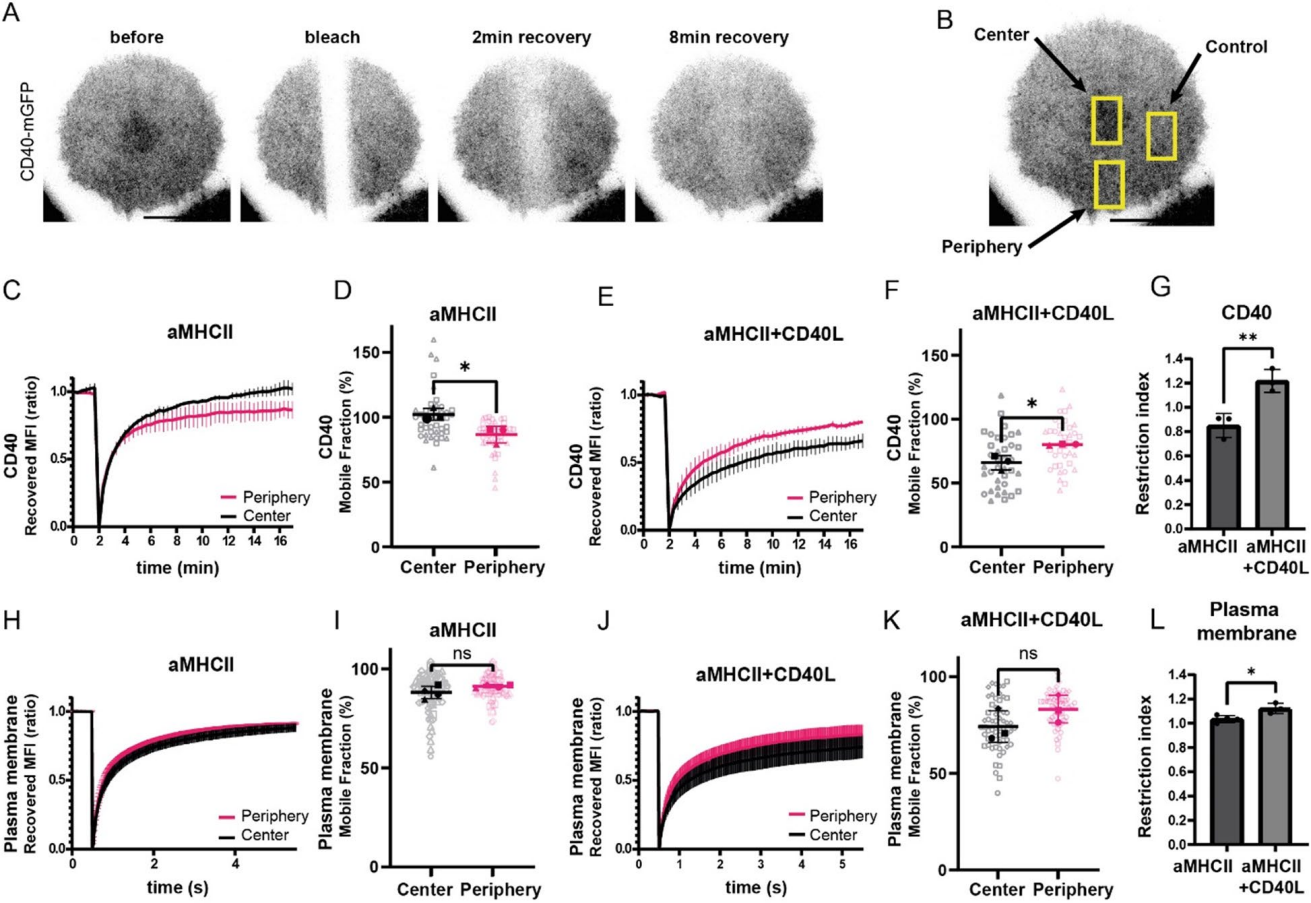
CD40 mobility is restricted by ligand engagement at the immunological synapse. **A** Representative time-lapse FRAP-TIRF images of a CD40-mGFP-expressing BMDC on an aMHCII + CD40L-coated surface, showing fluorescence before bleaching, immediately after bleaching, and at 2 and 8 min of recovery. Scale bar = 20 µm. **B** Schematic defining the peripheral and central regions of interest (ROIs) used for FRAP analysis. **C**-**G** FRAP analysis of CD40-mGFP mobility. **C** Fluorescence recovery time-course for CD40-mGFP on aMHCII-coated glass. **D** Mobile fraction of CD40-mGFP on aMHCII at 15 min post-bleach (n_1_ = 14 cells, n_2_ = 19 cells, n_3_ = 12 cells). **E** Fluorescence recovery time-course for CD40-mGFP on aMHCII + CD40L-coated glass. **F** Mobile fraction of CD40-mGFP on aMHCII + CD40L at 15 min post-bleach (n_1_ = 9 cells, n_2_ = 15 cells, n_3_ = 13 cells). **G** Restriction index for CD40 mobility, calculated from the mobile fractions in (**D**) and (**F**). **H**–**L** FRAP analysis of a fluorescent plasma membrane marker as a control. **H** Recovery time-course on aMHCII-coated glass. **I** Mobile fraction on aMHCII at 5 min post-bleach (n_1_ = 18 cells, n_2_ = 20 cells, n_3_ = 20 cells, n_4_ = 20 cells). **J** Recovery time-course on aMHCII + CD40L-coated glass. **K** Mobile fraction on aMHCII + CD40L at 5 min post-bleach (n_1_ = 21 cells, n_2_ = 18 cells, n_3_ = 21 cells). **L** Restriction index for the plasma membrane marker. For all graphs, data represent mean ± SD from three or four independent experiments (*n* = 3 and *n* = 4 for 3H,I) and statistical significance was determined using an unpaired Student’s t-test. *p < 0.05, **p < 0.01; ns, not significant

Our data suggested that CD40 clusters are centralized via a restriction of their diffusion. To test this hypothesis, we used a second light path coupled to a galvanometer scanner to perform fluorescence recovery after photobleaching on CD40-mGFP-expressing BMDCs imaged with TIRF microscopy (TIRF-FRAP) (Fig. 3A, Sup Movie 3). We bleached a rectangular band through the middle of the cell (Fig. 3B) and quantified the recovery of CD40-mGFP fluorescence at peripheral and central regions of the synapse, normalizing the intensity to a non-bleached control area to correct for photobleaching.

On surfaces coated with anti-MHCII alone, CD40 was highly mobile across the entire synapse, with the mobile fraction reaching nearly 100% in the center and 86% in the periphery after 15 min (Fig. 3C, D). However, upon CD40L engagement (on anti-MHCII + CD40L surfaces), the mobility of CD40 was drastically reduced specifically in the central cluster, with the mobile fraction only reaching 66% (Fig. 3E, F). In contrast, mobility in the periphery remained similarly high (80%). A restriction index, calculated by dividing the peripheral mobile fraction by the central mobile fraction, confirmed that CD40 diffusion is significantly constrained at the synapse center upon CD40L binding (Fig. 3G).

To determine if this mechanism operated globally on the plasma membrane, we repeated the TIRF-FRAP experiments on cells stained with the Cell Mask Deep Red plasma membrane dye (Sup Movie 4). We measured no significant difference in the mobile fraction of the plasma membrane between the center and the periphery, either in the presence or absence of CD40L (Fig. 3H, I, J, K). However, the restriction index revealed a small but significant reduction in general membrane mobility at the synapse center following CD40 triggering, which could result from molecular crowding due to CD40 accumulation (Fig. 3L). In conclusion, these experiments indicate that CD40 accumulation at the center of the synapse is mediated by a mechanism that specifically constrains its mobility, rather than a general change in membrane fluidity.

### CD40 centralization is driven by myosin IIA-dependent myosin contractions

The actin cytoskeleton at the dendritic cell immunological synapse is a critical organizer essential for T cell activation [42]. This aligns with the general principle that the cortical actomyosin cytoskeleton compartmentalizes the plasma membrane, creating barriers that restrict the diffusion of membrane proteins through physical corrals and myosin-driven contractility [26, 49, 55]. We therefore investigated how actin filaments and non-muscle myosin IIA contractions constrain the mobility of CD40 upon CD40L binding.

**Fig. 4:**
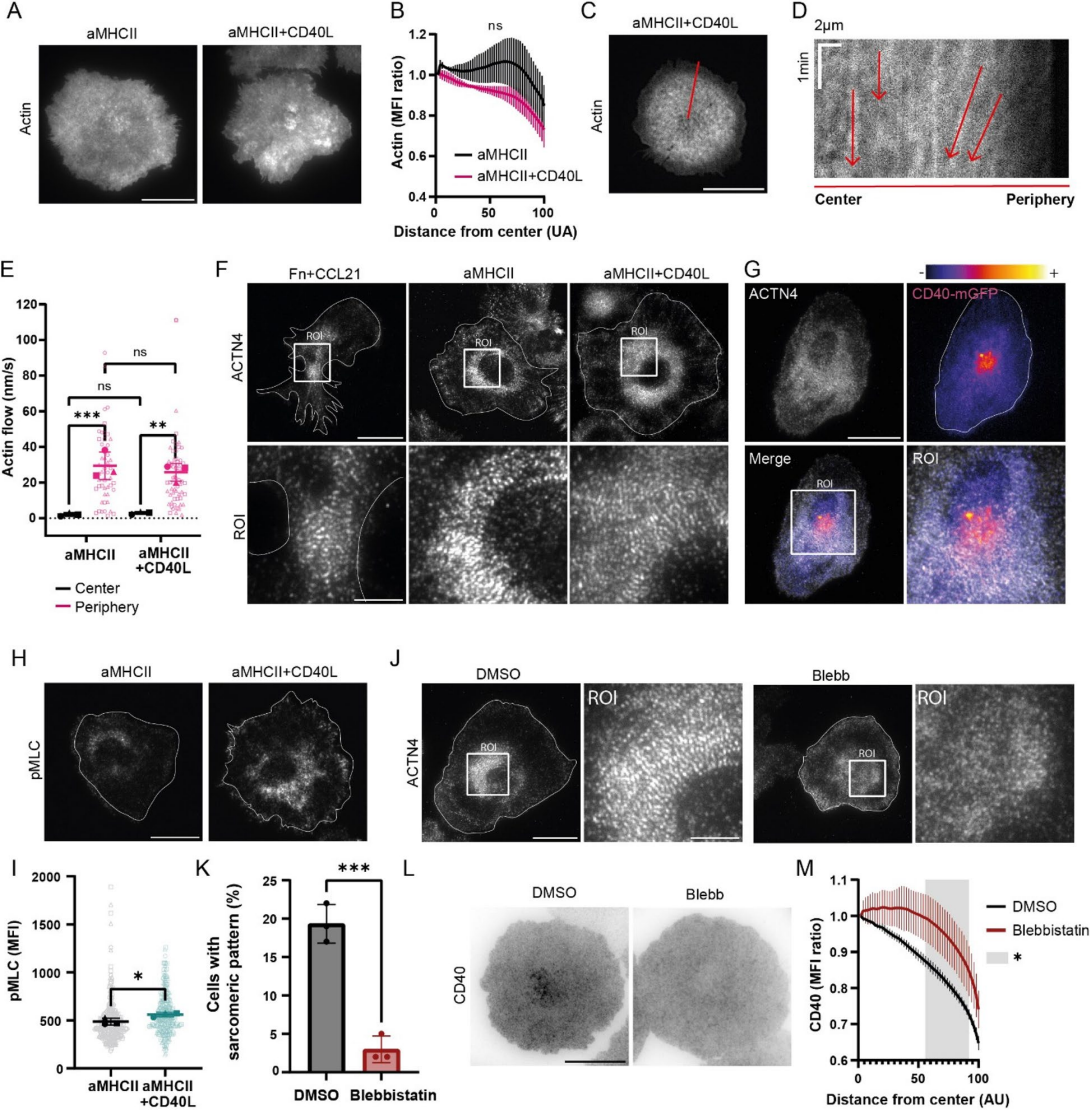
CD40 concentration at the synapse depends on actomyosin cytoskeleton activity. **A** Representative TIRF images of F-actin (phalloidin) in LPS-matured BMDCs on glass coated with anti-MHCII (aMHCII) or aMHCII + CD40L. **B** Quantification of radial actin mean fluorescence intensity (MFI), based on (A) (Two-way ANOVA, n_1_ = 82 cells, n_2_ = 97 cells, n_3_ = 98 cells). **C** Representative TIRF image of LifeAct-eGFP-BMDC on an aMHCII + CD40L-coated surface. The red line indicates the region used for the kymograph in (**D**). **D** Kymograph showing retrograde actin flow (indicated by arrows) from the region in (**C**). **E** Quantification of actin flow speed in the center versus the synapse’s periphery (Two-way ANOVA, n_1_ = 27 cells, n_2_ = 39 cells, n_3_ = 41 cells). **F** Representative TIRF images of α-actinin-4 on glass coated with Fibronectin and CCL21 (Fn + CCL21), aMHCII or aMHCII + CD40L, showing sarcomeric patterning in the ROIs. **G** Representative TIRF image showing co-localization of α-actinin-4 (grey) and CD40-mGFP (false-colored, fire LUT) on an aMHCII + CD40L-coated surface. **H** Representative TIRF images of phosphorylated myosin light chain (pMLC) staining. **I** Quantification of pMLC mean fluorescence intensity (MFI) (Unpaired Student’s t-test, n_1_ = 179 cells, n_2_ = 161 cells, n_3_ = 199 cells). **J** Representative TIRF images of α-actinin-4 in BMDCs treated with DMSO (vehicle) or 50 µM blebbistatin (Blebb). Insets show that blebbistatin treatment disrupts the sarcomeric pattern. **K** Quantification of the percentage of cells displaying a sarcomeric α-actinin-4 pattern on aMHCII + CD40L coated glass (mean of 3 blinded counters, Unpaired Student’s t-test, n_1_ = 251 cells, n_2_ = 267 cells, n_3_ = 239 cells). **L** Representative TIRF images of CD40 after DMSO or 50 µM blebbistatin treatment on aMHCII + CD40L coated glass. **M** Quantification of radial CD40 mean fluorescence intensity (MFI) after treatment (Two-way ANOVA, n_1_ = 45 cells, n_2_ = 49 cells, n_3_ = 46 cells). For all graphs, data are from three independent experiments (*n* = 3); bars represent mean ± SD. Main scale bar = 20 µm; inset scale bar = 10 µm. *p < 0.05, **p < 0.01, ***p < 0.001; ns, not significant

First, using TIRF microscopy, we imaged the cortical actin in fixed BMDCs and observed no obvious differences in actin organization or distribution with or without CD40L engagement (Fig. 4A), as confirmed by density profile quantification (Fig. 4B). To measure actin dynamics, we performed TIRF imaging and kymograph analysis on BMDCs expressing LifeAct-eGFP (Fig. 4C, D; Sup Movie 5). Quantification of these images revealed that actin flow was significantly faster at the periphery (25.75 nm/s ± 4.98 on aMHCII + CD40L) than at the center (2.93 nm/s ± 0.78 on aMHCII + CD40L) (Fig. 4E). However, CD40 triggering by immobilized CD40L did not influence these actin flow rates, indicating that global actin dynamics are unlikely to generate the central CD40 cluster.

Actomyosin arcs are known to compartmentalize the plasma membrane and restrict receptor diffusion. Concentric actomyosin arcs directly propel the inward transport of T cell receptor microclusters within the immunological synapse of T cells [38]. Additionally, α -actinin cross-linking, which contributes to structure these arcs, has been shown to limit the lateral diffusion of the B-cell antigen receptor [4]. We therefore examined the distribution of the actin cross-linking protein α-actinin-4 using TIRF microscopy of fixed BMDCs. In BMDCs plated on glass coated with Fn + CCL21, α-actinin-4 formed a periodic arrangement typical of sarcomeric patterns observed in motile non-muscle cells (Fig. 4F) [10]. Strikingly, upon synapse formation on anti-MHCII-coated glass, these sarcomeric structures rearranged to form concentric rings. The presence of CD40L did not affect these α-actinin-4 rings, but we observed that the central CD40 cluster was contained within them (Fig. 4G). As α-actinin can constrain the diffusion of ICAM-1 [15], we investigated its spatial relationship with the α-actinin-4 rings and found that ICAM-1 co-localized with the rings, independently of CD40 ligation (Sup Fig. 5A). Furthermore, the presence of ICAM-1 in the synapse neither induced CD40 centralization nor altered the extent of CD40 centralization in the presence of CD40L, indicating that ICAM-1 engagement is not required for CD40 spatial reorganization (Sup Fig. 5B). Together, these data demonstrate that CD40 centralization at the immune synapse is specifically driven by CD40L engagement and occurs independently of ICAM-1 ligation. These findings suggest that while α-actinin-4 ring structures organize the synapse and immobilize ICAM-1, an additional, distinct mechanism is responsible for CD40 centralization.

Although our data clearly localize CD40 within the α-actinin-4 rings, these structures are not modified by CD40 triggering and therefore cannot alone explain the formation of a central CD40 cluster. Myosin IIA is an integral component of actomyosin arcs, and its contractile activity is known to regulate receptor localization at the T cell immunological synapse [59, 61]. We investigated myosin activity by staining for phosphorylated myosin light chain (pMLC) and identified pMLC-rich rings that mirrored the α -actinin-4 structures (Fig. 4H). Crucially, pMLC staining was significantly brighter upon CD40-CD40L interaction, indicating that CD40 triggering increases myosin IIA activity at the synapse (Fig. 4I). To connect myosin IIA activity to the formation of the CD40 central cluster, we treated cells with 50 µM blebbistatin for 1 h before synapse formation and confirmed that this treatment did not affect cell viability nor the surface expression of MHCII and CD40 (Sup Fig. 6 A, B, C). Blebbistatin treatment led to the disorganization of α-actinin-4 in the sarcomeric rings, suppressing its periodic arrangement (Fig. 4J, K). Most importantly, the disruption of myosin IIA activity completely suppressed CD40 centralization upon CD40L binding (Fig. 4L), which was confirmed by a concentric density profile analysis (Fig. 4M). Crucially, these effects were not due to a general loss of synapse integrity, as blebbistatin did not inhibit overall cell spreading on the anti-MHCII surfaces. Moreover, while the ICAM-1 signal became more diffuse, mirroring the disorganization of the underlying α-actinin-4 rings, its fundamental association with these structures was preserved (Sup Fig. 6 D). To more carefully reassess the role of actin dynamics in actomyosin arc formation and CD40 positioning, we tested the effect of actin polymerization inhibitors. We used CK666 to inhibit branched actin formation [11, 24] and low dose of cytochalasin D (CytD) to inhibit retrograde actin flow [40, 59]. As expected, both treatments induced clear alterations in actin organization, leading to changes in synapse structure and spreading, thereby confirming the effective impact of these inhibitors on the actin cytoskeleton (Sup Fig. 6E, F, H, I, J). However, despite these pronounced structural perturbations, CD40 centralization within the synapse was preserved (Sup Fig. 6G, K). These results indicate that the defects in CD40 localization observed upon blebbistatin treatment cannot be explained solely by altered actin organization or actin flows, but instead specifically depend on inhibition of myosin II activity, highlighting the key role of actomyosin contractility in this process.

Altogether, our data identify contractile actomyosin rings at the immunological synapse that constrain the diffusion of CD40 into a central cluster. This process is mediated by an increase in myosin IIA activity following CD40L engagement, suggesting a feedback loop where CD40 signaling promotes cytoskeletal remodeling that, in turn, organizes and sustains receptor localization.

### CD40 centralization fine-tunes dendritic cell licensing

**Fig. 5:**
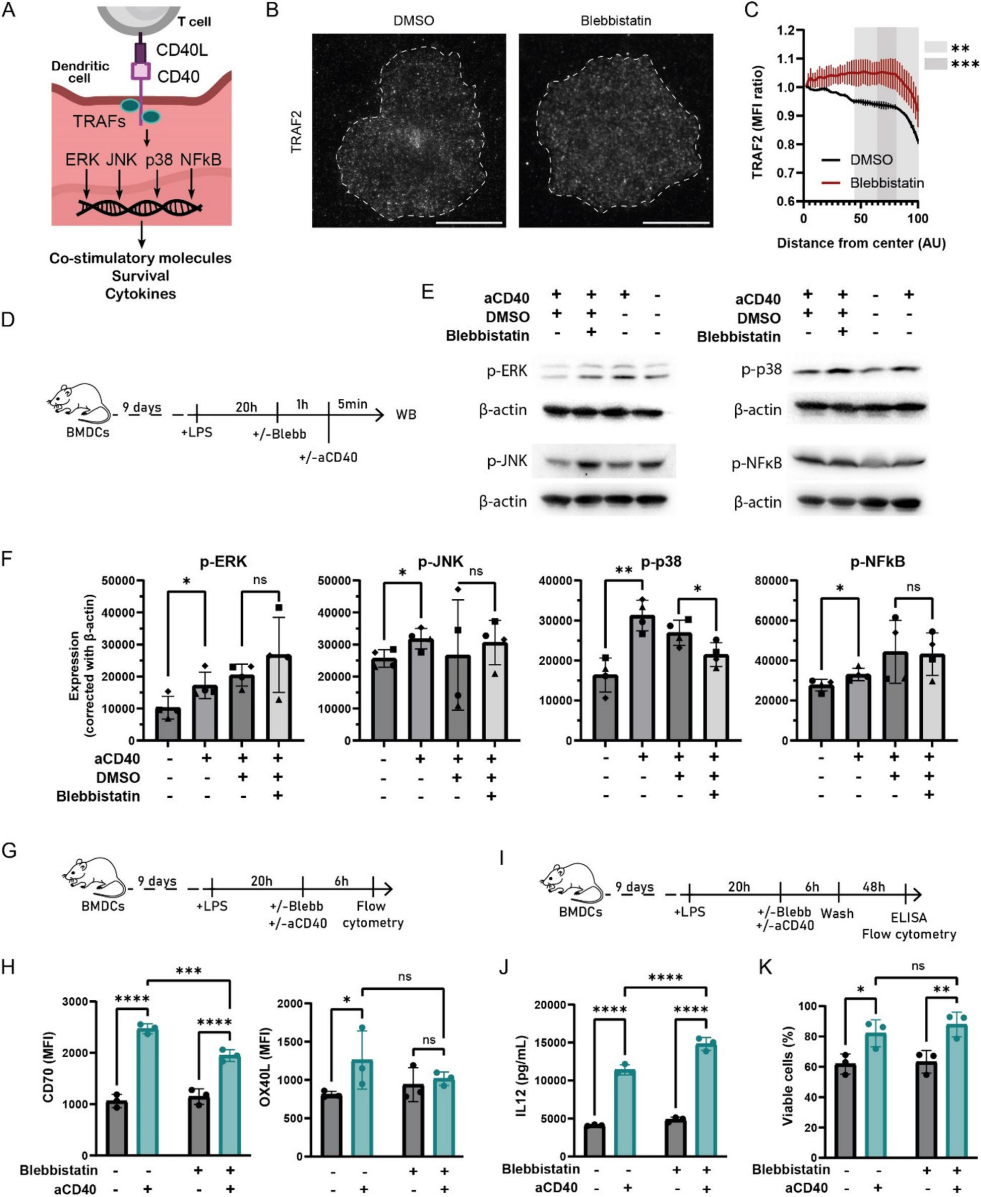
CD40 concentration at the synapse is required for fine-tuning of CD40-mediated dendritic cell licensing. **A** Schematic of CD40 downstream signaling upon CD40L ligation in licensing. **B** Representative TIRF images of TRAF2 after DMSO or 50 µM blebbistatin treatment on aMHCII + CD40L coated glass. Scale bar = 20 µm. **C** Quantification of radial TRAF2 mean fluorescence intensity (MFI) after treatment (Two-way ANOVA, n_1_ = 90 cells, n_2_ = 60 cells, n_3_ = 62 cells). **D** Experimental setup for in vitro recreation of CD40 signaling in BMDCs. LPS-matured BMDCs were pretreated with 50 µM blebbistatin or DMSO (vehicle) for 1 h at 37 °C, then stimulated with anti-CD40 (10 µg/mL) for 5 min to induce signaling. Cells were analyzed by western blot. **E** Representative western blots showing phosphorylation of ERK, JNK, p38 and NF-κB upon activation as described in (**D**) and β-actin as loading control. **F **Quantification of the western blots shown in (**E**) relative to β-actin (One-way ANOVA, symbols represent data from the same experiment). **G** Experimental setup to assess CD40 licensing function. LPS-matured BMDCs were treated with 50 µM blebbistatin or DMSO (control) and stimulated with anti-CD40 (10 µg/ml) for 6 h at 37 °C. **H** Quantification of CD70 and OX40L expression (median fluorescence intensity, MFI) by flow cytometry after stimulation as described in (**G**) (Two-way ANOVA). **I** Experimental setup to assess CD40 licensing function. LPS-matured BMDCs were treated with 50 µM blebbistatin or DMSO (control) and stimulated with anti-CD40 (10 µg/mL) for 6 h at 37 °C. Cells were washed and cultured for 48 h before assessing IL-12 release and cell viability. **J** Quantification of IL-12 release in the 48 h culture supernatant (Two-way ANOVA). **K** Quantification of BMDC survival assessed using Sytox Blue live/dead staining (Two-way ANOVA). For all graphs, data represent mean ± SD from three or four independent experiments (*n* = 3 and *n* = 4 for 5E,F). *p < 0.05, **p < 0.01, ***p < 0.001, ****p < 0.0001; ns, not significant

CD40 plays a central role in the licensing of mature dendritic cells, upgrading their capacity to prime CD8^+^ T cells by upregulating co-stimulatory molecules and cytokines [16, 47]. This process is driven by the recruitment of TRAFs proteins and the activation of MAP Kinases, specifically p38 MAPK, JNK and ERK, which, together with transcription factors such as NF-κB, lead to the subsequent production of various cytokines such as IL-6, IL-12 and TNF-α [36] and the upregulation of several co-receptors including CD70 [9] or OX40L [37]. Activation of CD40 signaling further promotes dendritic cell survival [57] (Fig. 5A). We therefore sought to determine how CD40 concentration at the synapse influences its downstream signaling. To do so, we used myosin inhibition with blebbistatin, which disrupted the CD40 central cluster without affecting synapse formation or ICAM-1 anchorage to the contractile rings, and then assessed key licensing outcomes. We first confirmed that recruitment of TRAF2 to the center of the synapse was inhibited in blebbistatin-treated BMDCs, thus further demonstrating that CD40 activity could be impaired by inhibition of its centralization (Fig. 5B, C).

Western blot analysis confirmed that CD40 ligation successfully induced the phosphorylation of ERK, JNK, p38 and NF-κB (Fig. 5C, D). However, we observed that the inhibition of CD40 centralization by blebbistatin specifically and significantly reduced the activation of the p38 MAPK pathway, while other pathways remained unaffected (Fig. 5C, D). Importantly, to control for potential off-target effects of blebbistatin on signaling pathways, we independently assessed ERK phosphorylation downstream of CCL19-CCR7 stimulation. Blebbistatin treatment did not alter CCL19-induced ERK activation, indicating that blebbistatin does not globally impair MAPK signaling and supporting the specificity of its effects on CD40 signaling (Sup Fig. 7A, B).

We next assessed the functional consequences of this altered signaling after 6 h of CD40 licensing (Fig. 5E). Consistent with reduced p38 MAPK signaling, we found that the upregulation of the co-stimulatory ligands CD70 and OX40L was impaired in blebbistatin-treated cells (Fig. 5F). Interestingly, disrupting the CD40 central cluster potentiated IL-12 secretion, while cell survival was not affected after 48 h (Fig. 5G, H, I). These findings are consistent with the suppressive effect of p38 on IL-12 observed in mouse dendritic cells [58], contrasting with previous reports of its stimulatory role in human [1, 60]. Mechanistically, this effect can be explained by the impaired recruitment and centralization of TRAF2 under blebbistatin treatment, as TRAF2 recruitment to CD40 cytoplasmic tail after engagement of CD40 is necessary for p38 activation and can impact subsequent IL-12 production [31, 54]. Altogether, these results suggest that the formation of a central CD40 cluster following its ligation fine-tunes downstream signaling pathways. This specific regulation highlights a key role for the spatial organization of CD40 receptors in modulating the licensing capacity of dendritic cells.

## Discussion

The engagement of CD40 on dendritic cells by CD40L on T cells is critical to the communication between these two cell types and a central event in the establishment of adaptive immunity. Yet, how the spatial organization of this receptor-ligand pair within the immunological synapse dictates the functional outcome of dendritic cell licensing remains poorly understood. In this study, we used the BMDC model on engineered surfaces. This strategy enabled the use of high spatial and temporal resolution microscopy techniques to uncover fundamental principles of receptor-cytoskeleton interactions at the immunological synapse of dendritic cells. Our data show that the nanoscale distribution of CD40 is dynamically remodeled during dendritic cell maturation and synapse formation. We propose a mechanism whereby CD40L engagement triggers the myosin IIA-dependent contraction of actomyosin rings to promote the formation of a central CD40 cluster. This spatial reorganization is not merely structural but appears to be critical for fine-tuning downstream signaling pathways that orchestrate dendritic cell licensing.

The prevailing model for the activation of TNF superfamily (TNFSF) members involves a two-step process: initial ligand-induced trimerization of the receptor followed by the formation of higher-order oligomers, or "super-clusters," which are required for robust signal transduction [51]. This clustering increases the local concentration of receptors, enhancing the recruitment of low-affinity downstream adaptors like TRAFs and amplifying the signaling cascade [19, 28]. Therefore, the regulation of receptor distribution on the cell surface is a key mechanism for modulating their activity. Our findings align with this principle, as we detected pre-formed nanoclusters of CD40 on the surface of immature dendritic cells. However, we observed that upon maturation with LPS, these clusters disassemble, leading to smaller, more numerous aggregates despite an overall increase in CD40 expression.

This seemingly counterintuitive de-clustering upon receiving a pro-inflammatory maturation signal has precedents in other receptor systems where dispersal is a key regulatory step. For instance, in resting B cells, B-cell receptors (BCRs) are confined in nanoclusters by the actin cytoskeleton; upon stimulation, actin remodeling leads to their dispersal, which increases their mobility and facilitates interactions with co-receptors, ultimately amplifying the signal [48]. Similarly, the dispersal of acetylcholine receptor (AChR) clusters at the neuromuscular junction is an active, cytoskeleton-dependent process that regulates synaptic plasticity [2]. We propose that the de-clustering of CD40 serves a dual purpose for the maturing dendritic cell. First, it likely raises the activation threshold. By dispersing CD40, the dendritic cell reduces the receptor’s apparent avidity for CD40L, ensuring that licensing occurs only in response to stable, high-avidity interactions with cognate T cells in the lymph node. Second, dispersal may liberate smaller, more mobile CD40 units, priming them for rapid reorganization into a newly forming synapse. Consistent with this idea, our TIRF-FRAP experiments (Fig. 3) demonstrate that CD40 exhibits unusually high mobility under basal conditions, which becomes constrained upon ligand engagement at both the center and periphery of the synapse. Upon synapse formation, these smaller clusters are further restricted, within a ring of ICAM-1. This suggests a model where a large pool of CD40 is maintained in a mobile, yet quiescent, state through de-clustering, ready to be rapidly concentrated into a supramolecular cluster (SMAC), consistent with a lateral redistribution mechanism for potent activation within the adhesive ring of the dendritic cell-T cell interface.

The formation of this central CD40 cluster appears to be largely driven by the underlying actomyosin cytoskeleton. The cortical cytoskeleton is known to create diffusion barriers that compartmentalize the plasma membrane [49], and this is often a highly active process. In T cells, concentric actomyosin arcs, structured by the cross-linker α-actinin, actively propel TCR microclusters toward the synapse center [38]. This provides a clear example of how biophysical forces generated by actomyosin contractions are directly linked to the regulation of receptor signaling. We identified a pre-established cytoskeletal architecture at the dendritic cell synapse, composed of concentric rings of α-actinin-4, ICAM-1, and active myosin IIA. This structure organizes ICAM-1 upon synapse formation but appears to only constrain CD40 after it is triggered by CD40L. This ligation event is associated with a significant increase in myosin IIA activity, which drives the contraction of the rings and plays a major role in the subsequent formation of the central CD40 cluster. This suggests a dual function for the actomyosin ring: it first establishes a stable adhesive zone by organizing ICAM-1, and then, upon a specific activation signal from CD40, it transforms into a contractile machine that actively organizes CD40 within that zone. Interestingly, our data show that CD40 forms a central cluster at the immunological synapse while its ligand, CD40L, is immobilized on a rigid surface in our model. This led us to propose a “kiss-run-and-trap” mechanism, in which CD40 first engages immobilized CD40L at the synapse periphery, triggering signaling (“kiss”), then diffuses across the membrane (“run”) until it is corralled into the central cluster (“trap”) by the actomyosin ring.

Mechanistically, these findings are consistent with a model in which CD40 engagement activates the RhoA-ROCK signaling pathway. In B cells, CD40 stimulation induces the expression of the RhoA guanine nucleotide exchange factor p190RhoGEF, leading to RhoA activation, cytoskeletal remodeling, and NF-κB-dependent differentiation programs [23, 29]. Although this pathway has not been directly examined in dendritic cells, it provides a plausible mechanistic framework linking CD40 ligation to actomyosin contractility. In this model, CD40-dependent RhoA activation could engage ROCK to promote myosin II activation through increased myosin light chain phosphorylation, thereby generating the actomyosin-dependent forces required for CD40 centralization at the synapse.

This signal-dependent trapping mechanism can be viewed as a form of molecular clutch, where CD40 is engaged by the contractile machinery only after it binds its ligand. In dendritic cells, the tethering of ICAM-1 to the actin cortex via α-actinin and ERM proteins (ezrin, radixin and moesin) is known to constrain its mobility, providing a stable anchor for T cell LFA-1 to pull against [15]. Our data build on this by showing that this same structure serves as a dynamic scaffold for another receptor, CD40. The increase in myosin IIA activity upon CD40 ligation likely increases local tension and stiffens the ring structure, transforming it into an effective diffusion barrier specifically for engaged CD40 molecules, thereby promoting their accumulation. Notably, endosomal trafficking has been shown to play a crucial role in T cell synapse formation [21]. The visible intracellular pool of CD40 in our conjugates suggests that vesicular trafficking pathways, such as endocytosis and polarized recycling, could additionally contribute to CD40 centralization alongside lateral motility. Looking forward, further investigations into potential CD40 recycling at the synapse could provide additional insight into the mechanisms that regulate synapse formation and CD40 receptor trafficking, offering a broader understanding of how dendritic cells control CD40 spatial organization.

Finally, the spatial organization of receptors within the synapse is known to fine-tune signaling outcomes. For example, the centralization of TCRs into the central SMAC is associated with signal termination and receptor downregulation [53]. The formation of large receptor aggregates can alter the recruitment of signaling effectors, with higher-order clusters being necessary to recruit low-affinity adaptors and initiate specific downstream pathways [19]. We found that preventing the formation of the central CD40 cluster via myosin inhibition leads to a selective fine-tuning of downstream signaling. While the activation of NF-κB, ERK, and JNK pathways was unaffected, the p38 MAPK pathway was significantly impaired, resulting in reduced upregulation of the costimulatory molecules CD70 and OX40L and modification of IL-12 secretions. This is in line with the observed inhibition of TRAF2 recruitment, which has been shown to be necessary for CD40-driven p38 activation and tuning of IL-12 production [31, 54]. Furthermore, while CD40 ligation is a potent inducer of IL-12 production [50], the magnitude of this pro-inflammatory response is critical for determining the subsequent T cell fate. Graded exposure to inflammatory cytokines like IL-12 directs the differentiation of CD8 + T cells into either short-lived effector cells or long-term memory precursors, highlighting the need to precisely control these signals to ensure effective immunity without causing immunopathology [27]. In this context, it was striking that while some licensing outputs were diminished upon myosin inhibition IL-12 secretion was potentiated.

This indicates that the spatial organization of CD40 acts as a molecular rheostat, not a simple on/off switch. The formation of a dense central cluster appears to be required for the efficient activation of the p38-dependent licensing pathway leading to the expression of key co-stimulatory molecules. In contrast, a more dispersed configuration of CD40, as seen during myosin inhibition, may favor pathways leading to IL-12 production. This suggests a model where the dendritic cell can modulate the quality of its licensing response based on the biophysical properties of the synapse. For instance, the high CD70/OX40L and moderate IL-12 phenotype driven by a central CD40 cluster may be geared towards priming robust, terminally differentiated effector T cells. In contrast, the alternative state induced by dispersed CD40, characterized by lower co-stimulation but high IL-12, might favor the differentiation of T cells with a different functional profile, as the specific balance of these signals is known to program distinct T cell fates. This suggests a model where the dendritic cell can modulate the quality of its licensing response based on the biophysical properties of the synapse.

## Conclusions

In summary, our study reveals a complex interplay between receptor nanoscale organization, cytoskeletal mechanics, and signal transduction, where myosin IIA-driven contractility is critical for the spatial organization of CD40, which, potentially alongside intracellular trafficking mechanisms orchestrates the precise functional outcomes of dendritic cell licensing.

## Supplementary Information

Supplementary Material 1.

Supplementary Material 2.

Supplementary Material 3.

Supplementary Material 4.

Supplementary Material 5.

Supplementary Material 6.

## Data Availability

Data supporting this study’s conclusions have been deposited in Zenodo, http://doi.org/10.5281/zenodo.17021860. Due to their volume, some microscopy image datasets are only available from the authors upon reasonable request and will be shared freely.

## Abbreviations

AChR: Acetylcholine receptor
BCR: B-cell receptor
BMDC: Bone marrow-derived dendritic cell
BSA: Bovine serum albumin
CD40L: CD40 ligand
DMSO: Dimethyl sulfoxide
dSTORM: Direct stochastic optical reconstruction microscopy
EGFP: Enhanced green fluorescent protein
eps: Epsilon (DBSCAN clustering parameter)
ERK: Extracellular signal-regulated kinase
ERM: Ezrin, Radixin, Moesin
Fn: Fibronectin
FRAP: Fluorescence recovery after photobleaching
GM-CSF: Granulocyte-macrophage colony-stimulating factor
HEPES: 4-(2-Hydroxyethyl)-1-piperazineethanesulfonic acid
ICAM-1: Intercellular adhesion molecule 1
iFCS: Inactivated fetal calf serum
IL-12: Interleukin 12
IL-7: Interleukin 7
JNK: C-Jun N-terminal kinase
LPS: Lipopolysaccharide
MAPK: Mitogen-activated protein kinase
MF: Mobile fraction
MHC: Major histocompatibility complex
minPts: Minimum points (DBSCAN clustering parameter)
MOI: Multiplicity of infection
NF-κB: Nuclear factor kappa B
ns: Not significant
OVA: Ovalbumin
PBS: Phosphate-buffered saline
PLAD: Pre-ligand-binding assembly domain
PLL: Poly-L-Lysine
R10: RPMI 1640 supplemented culture medium
RI: Restriction index
ROI: Region of interest
SMAC: Supramolecular activation cluster
TCR: T cell receptor
TIRF: Total internal reflection fluorescence
TNFSF: Tumor necrosis factor superfamily
TRAF: TNF receptor-associated factor
VSVg: Vesicular stomatitis virus glycoprotein
α-actinin: Alpha-actinin
