# Supplementary material for: Actomyosin rings constrain CD40 mobility to organize the dendritic cell immunological synapse
Source: Cell Commun Signal. 2026 Apr 18;24:326. doi: 10.1186/s12964-026-02816-0 (PMC13220446; doi:10.1186/s12964-026-02816-0)
Supplement: Supplementary file 6 — Supplementary Material 6. [file 12964_2026_2816_MOESM6_ESM.pdf]

Supplementary Movie 1. Live-cell TIRF imaging of CD40-mGFP dynamics at the synapse. Time-lapse TIRF microscopy of an LPS-matured BMDC expressing CD40-mGFP, seeded onto anti-MHCII+CD40L (aMHCII+CD40L) coated glass. The video shows real-time accumulation and redistribution of CD40 at the immune synapse. Timestamp displayed as hh:mm. Scale bar = 20 μm. Representative of n=3 independent experiments (biological replicates)

Supplementary Movie 2. 3D representation of CD40 recruitment to the synapse. Representative confocal 3D reconstruction of interactions between OT-II T cells and BMDCs primed with OVA peptide. Cells are stained for tubulin (magenta) and CD40 (cyan, BMDCs).

Supplementary Movie 3. Representative FRAP-TIRF time-lapse imaging of CD40-mGFP in BMDCs on aMHCII+CD40L. Time-lapse sequence showing fluorescence recovery after photobleaching (FRAP) in an LPS-matured BMDC expressing CD40-mGFP, seeded on anti-MHCII+CD40L (aMHCII+CD40L) coated glass. Images were acquired using TIRF microscopy. The photobleached region and subsequent fluorescence recovery illustrate CD40 dynamics at the synapse. Scale bar = 20 μm. Representative of n=3 independent experiments (biological replicates)

Supplementary Movie 4. Representative FRAP-TIRF time-lapse imaging of CD40-mGFP in BMDCs on aMHCII. Time-lapse sequence showing fluorescence recovery after photobleaching (FRAP) in a LPS-matured BMDC expressing CD40-mGFP, seeded on anti-MHCII (aMHCII) coated glass. Images were acquired using TIRF microscopy. The photobleached region and subsequent fluorescence recovery illustrate CD40 dynamics at the synapse. Scale bar = 20 μm. Representative of n=3 independent experiments (biological replicates)

Supplementary Movie 5. Actin dynamics visualized with LifeAct-eGFP at the synapse. Time-lapse TIRF imaging of an LPS-matured BMDC expressing LifeAct-eGFP on anti-MHCII+CD40L coated glass. This video captures retrograde actin flow within the immune synapse. Scale bar = 10 μm. Representative of n=3 independent experiments (biological replicates)

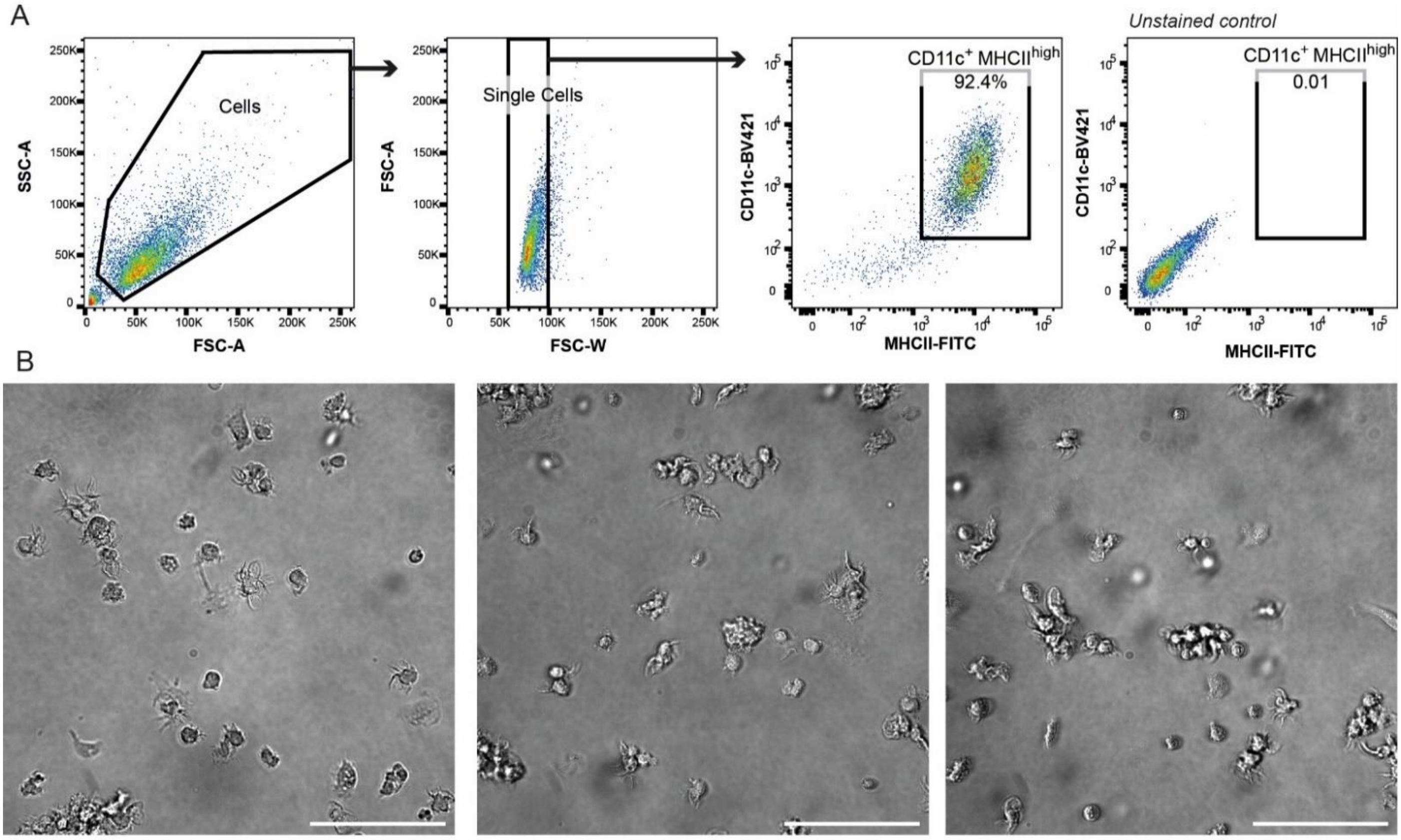

Supplementary Figure 1. Characterization of bone marrow–derived dendritic cell (BMDC) populations. A. Gating strategy showing our culture of BMDCs are composed of $CD11c^{+}$ $MHCII^{high}$ cells. **B.** Bright field images of BMDCs cultures. Scale Bar = 100μm. Data representative of all of BMDCs cultures.

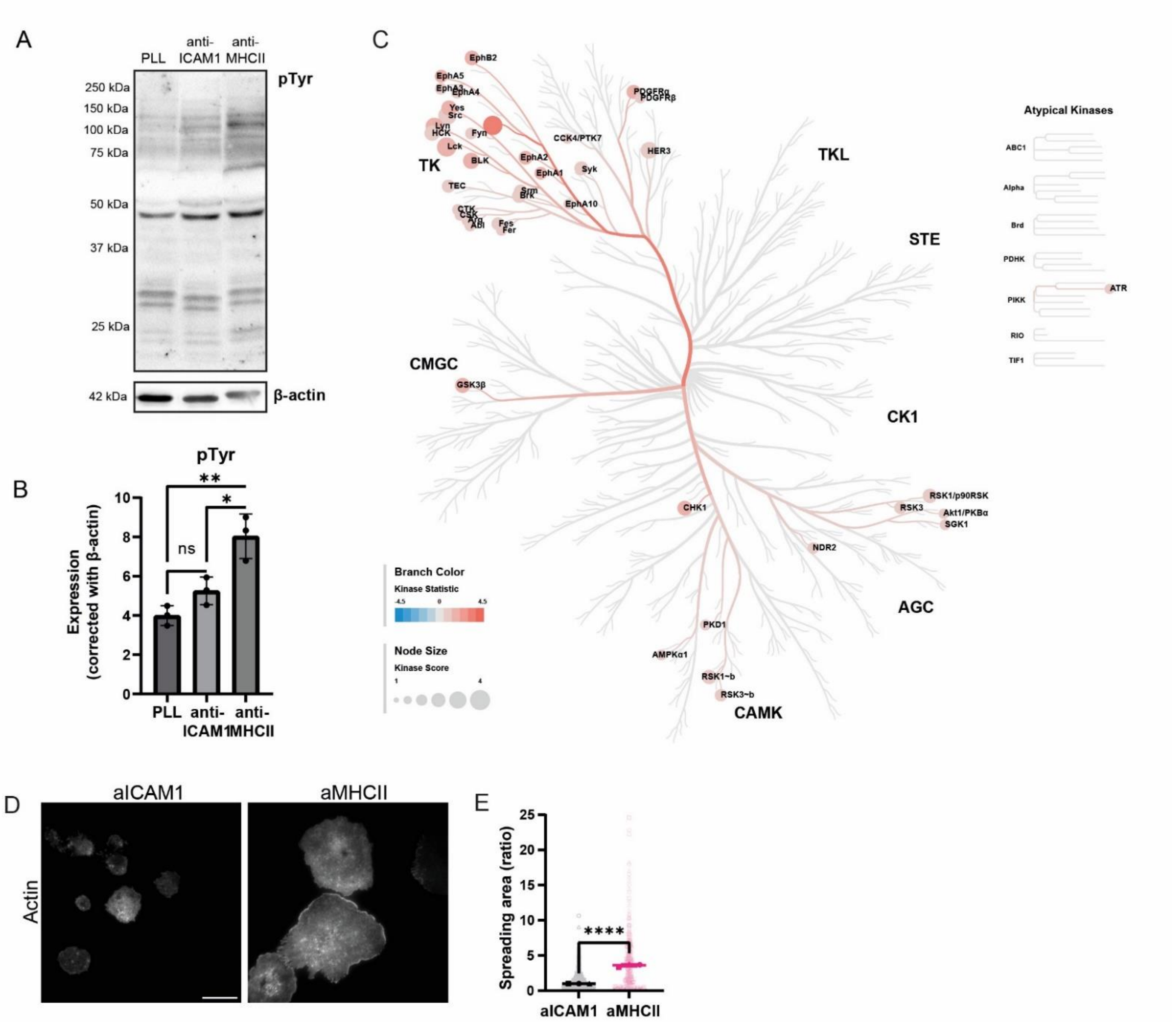

Supplementary Figure 2. Anti-MHCII coating induces a cellular activation and pseudo synapse formation. **A.** Representative western blots showing tyrosine phosphorylations (pTyr) in BMDCs upon activation with PLL, anti-ICAM1 or anti-MHCII coated beads, β-actin as loading control. Cells were activated with coated beads at a ratio of 1:1 for 5min. **B**. Quantification of western blot data from (A), showing normalized levels of tyrosines relative to β-actin (One-way ANOVA, n=3). **C.** Kinome Coral Tree highlighting protein tyrosine kinases that were upregulated in anti-MHCII-beads treated samples compared to PLL-beads after 5 min of activation. Branch color corresponds to the direction of effect: the change in kinase activity in the anti-MHCII condition compared to the control (< 0 means inhibition, > 0 means activation in the treated condition). Node Size corresponds to significance. The tree only display Kinase Score > 1.3. (n=1, cells from 3 independent mice and BMDCs cultures were used in a single technical replicate). **D**. Representative TIRF images of F-actin (phalloidin) of mature BMDCs on anti-ICAM-1 (aICAM1) or anti-MHCII (aMHCII) coated glass, after 30min of cell adhesion. Scale bar = 20μm **E**. Quantification of the BMDCs spreading area from (D), normalized to their area on aICAM1 (unpaired Student's t-test, n_1=347 cells, n_2=327 cells, n_3=304 cells). Bars represent mean ± SD from three independent experiments (n=3). *p < 0.05, **p < 0.01, ***p < 0.001, ****p < 0.0001; ns, not significant.

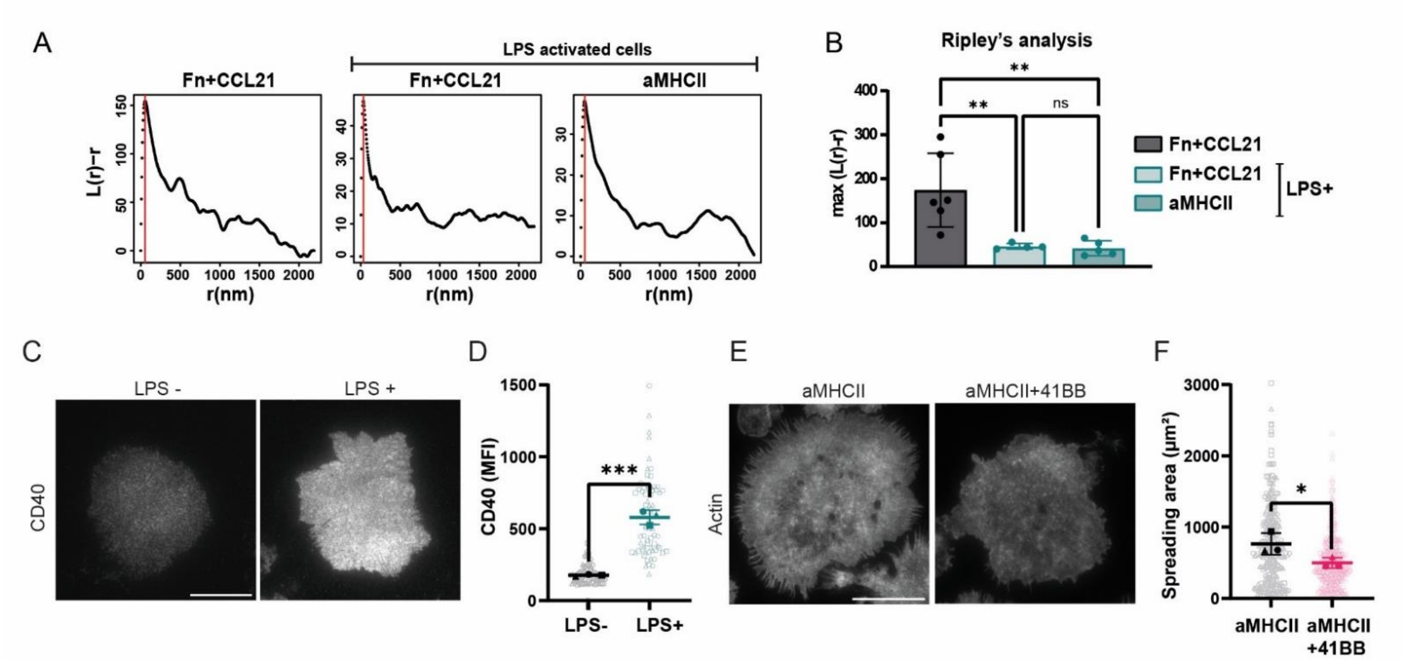

Supplementary Figure 3. TNFSF clustering and expression, and synapse size depend on BMDC maturation and TNFSF presence. **A**. Representative Ripley's L(r)-r functions from dSTORM microscopy acquisition of CD40-stained BMDCs on Fibronectin+CCL21 (Fn+CCL21) or aMHCII coated glass. When indicated, BMDCs were matured overnight with LPS. **B**. Quantification of Ripley's functions (L(r)-r function) using dSTORM (each dot corresponds to one cell). **C**. Representative TIRF images of CD40 stained BMDCs on aMHCII coated glass. **D**. Quantification of the CD40 mean fluorescence intensity (MFI) on the glass (n_1=54 cells, n_2=45 cells, n_3=51 cells). **E**. Representative TIRF images of phalloidin stained BMDCs on anti-MHCII or anti-MHCII and 41BB coated glass (aMHCII+41BB). **F**. Quantification of the BMDCs spreading area on the glass (n_1=250 cells, n_2=196 cells, n_3=275 cells). For all graphs, data were collected in three independent experiments (n=3), bars indicate mean ± SD. Statistical significance was determined using an Unpaired Student's t-test. *$p < 0.05$, **$p < 0.01$, ***$p < 0.001$; ns, not significant. Scale bars = 20µm

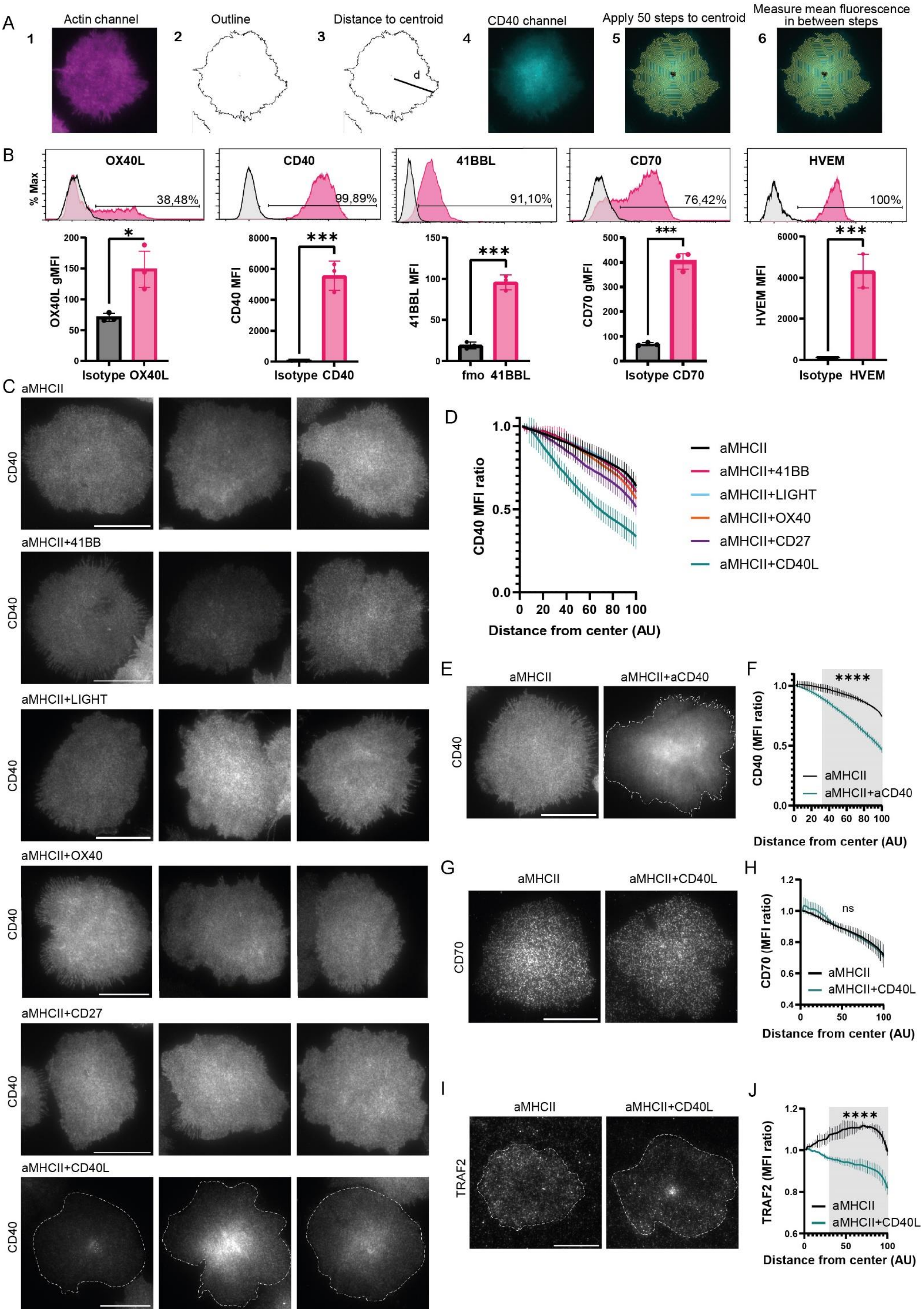
A
1 Actin channel
2 Outline
3 Distance to centroid
d
4 CD40 channel
5 Apply 50 steps to centroid
6 Measure mean fluorescence in between steps
B
% Max
OX40L 38,48%
CD40 99,89%
41BBL 91,10%
CD70 76,42%
HVEM 100%
OX40L gMFI
Isotype OX40L
CD40 MFI
Isotype CD40
41BBL MFI
fmo 41BBL
CD70 gMFI
Isotype CD70
HVEM MFI
Isotype HVEM
C
aMHCII
aMHCII+41BB
aMHCII+LIGHT
aMHCII+OX40
aMHCII+CD27
aMHCII+CD40L
CD40
D
CD40 MFI ratio
Distance from center (AU)
aMHCII
aMHCII+41BB
aMHCII+LIGHT
aMHCII+OX40
aMHCII+CD27
aMHCII+CD40L
E
aMHCII
aMHCII+aCD40
CD40
F
CD40 (MFI ratio)
****
aMHCII
aMHCII+aCD40
Distance from center (AU)
G
aMHCII
aMHCII+CD40L
CD70
H
CD70 (MFI ratio)
ns
aMHCII
aMHCII+CD40L
Distance from center (AU)
I
aMHCII
aMHCII+CD40L
TRAF2
J
TRAF2 (MFI ratio)
****
aMHCII
aMHCII+CD40L
Distance from center (AU)

Supplementary Figure 4. Impact of co-stimulatory ligands on spatial distribution of CD40, CD70 and TRAF2 in BMDCs. A. Radial quantification workflow. Shortly, the cell outline was determined using the actin (1-2). Using the distance (d) from the outline to centroid (3), the outline was reduced step-by-step to the center of the cell, 50 steps total (4-5). The mean fluorescence intensity (MFI) of CD40 was quantified in between steps (6). **B.** Surface expression of TNFSF members on BMDCs. Median (MFI) or geometric mean (gMFI) intensity of fluorescence is compared to isotype or to fluorescence minus one (fmo) when an isotype wasn't available, with their respective representative histograms and percentages of positives cells (Unpaired Student's t-test, n=3). **C.** Representative TIRF images of CD40 on mature BMDCs on anti-MHCII (aMHCII) or anti-MHCII supplemented with 41BB , LIGHT, OX40, CD27 or CD40L, (n=1 biological replicate). **D.** Quantification of radial CD40 mean fluorescence intensity (MFI) from (C) (Two-way ANOVA, n_{aMHCII}=16 cells, n_{41BB}=6 cells, n_{OX40}=12 cells, n_{CD27}=11 cells, n_{CD40L}=10 cells). **E.** Representative TIRF images of CD40 on aMHCII or aMHCII+anti-CD40 (aCD40) coated glass. **F**. Quantification of radial CD40 MFI on aMHCII or aMHCII+aCD40 coated glass (Two-way ANOVA, n1=62 cells, n2=26 cells, n3=22 cells). **G.** Representative TIRF images of CD70 on aMHCII or aMHCII+CD40L coated glass. **H**. Quantification of radial CD70 MFI on aMHCII or aMHCII+CD40L coated glass (Two-way ANOVA, n1=72 cells, n2=65 cells, n3=62 cells). **I.** Representative TIRF images of TRAF2 on aMHCII or aMHCII+CD40L coated glass. **J**. Quantification of radial TRAF2 MFI on aMHCII or aMHCII+CD40L coated glass (Two-way ANOVA, n_1=70 cells, n_2=68 cells, n_3=86 cells). For all graphs, if not indicated otherwise, data were collected in three independent experiments (n=3), bars indicate mean ± SD. *p < 0.05, **p < 0.01, ***p < 0.001, ****p<0.0001; ns, not significant. Scale bars = 20μm

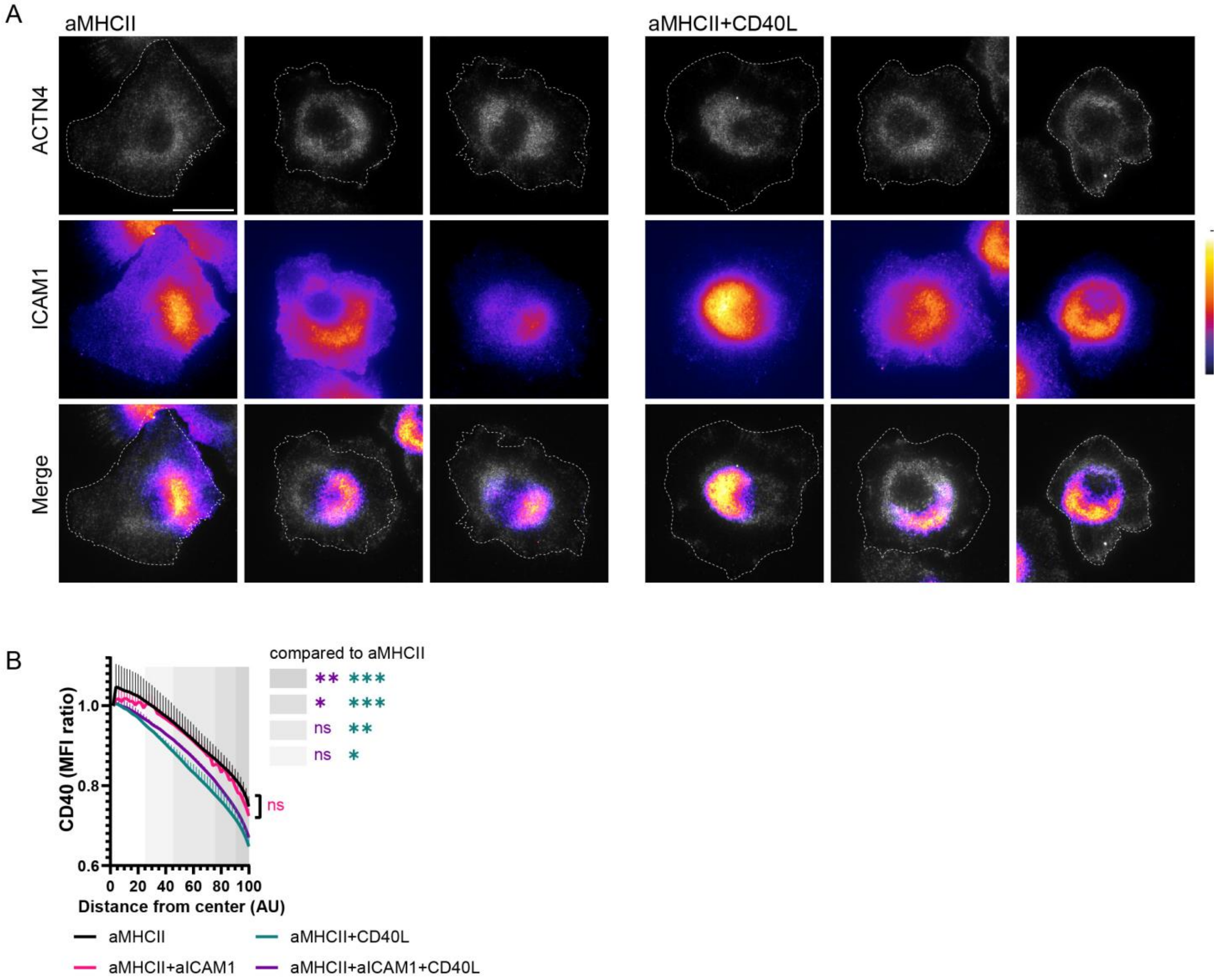

Supplementary Figure 5. ICAM-1 localization to the α-actinin ring is independent of CD40–CD40L engagement and does not affect synaptic CD40 concentration. A. Representative TIRF image showing co-localization of α-actinin-4 (grey) and ICAM-1 (false-colored, fire LUT) of a BMDC on an anti-MHCII (aMHCII) or aMHCII+CD40L-coated surface. Scale bar = 20μm **B.** Quantification of radial CD40 mean fluorescence intensity (MFI) from BMDCs on anti-MHCII (aMHCII) supplemented when indicated with anti-ICAM1 (aMHCII+aICAM1), CD40L (aMHCII+CD40L), anti-ICAM1s and CD40L (aMHCII+aICAM1+CD40L) (Two-way ANOVA compared to aMHCII, n_1=166 cells, n_2=202 cells, n_3=163 cells)

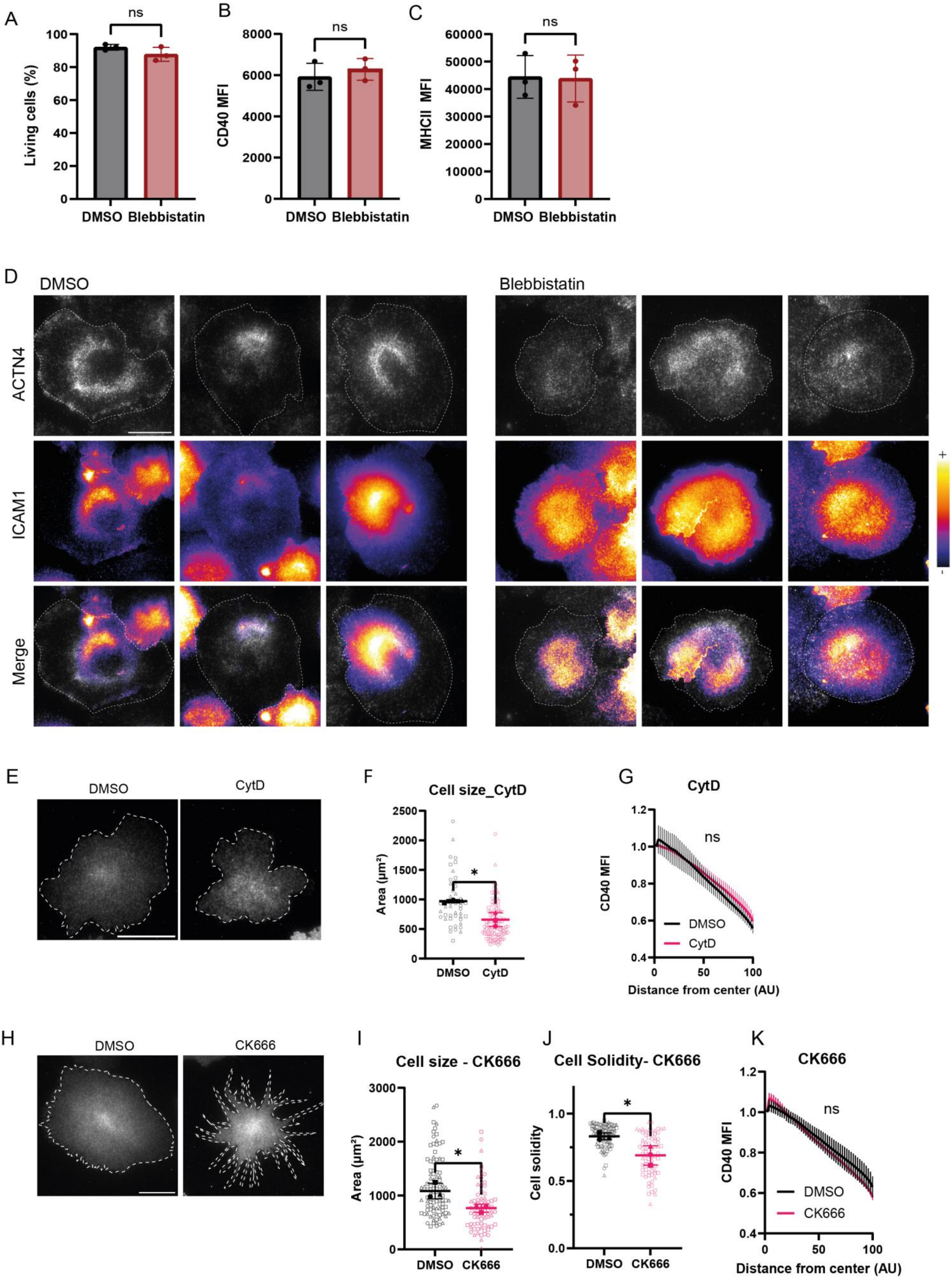
A
ns
Living cells (%)
DMSO Blebbistatin
B
ns
CD40 MFI
DMSO Blebbistatin
C
ns
MHCII MFI
DMSO Blebbistatin
D
DMSO
Blebbistatin
ACTN4
ICAM1
Merge
E
DMSO
CytD
F
Cell size_CytD
Area (µm²)
DMSO CytD
G
CytD
ns
CD40 MFI
DMSO
CytD
Distance from center (AU)
H
DMSO
CK666
I
Cell size - CK666
Area (µm²)
DMSO CK666
J
Cell Solidity- CK666
Cell solidity
DMSO CK666
K
CK666
ns
CD40 MFI
DMSO
CK666
Distance from center (AU)

Supplementary Figure 6. Cytoskeletal inhibitors differentially affect BMDC synapses. A-C. Blebbistatin treatment does not impact BMDCs survival, CD40 and MHCII surface expression. LPS-matured BMDCs were treated with 50μM blebbistatin or DMSO (control) for 1 h at 37 °C before being stained for flow cytometry. (A) Quantification of BMDC survival using Sytox Green live/dead staining, (B) CD40 and (C) MHCII surface expression (median fluorescence intensity, MFI) assessed by flow cytometry (Unpaired Student's t-test, n=3). **D.** Representative TIRF image showing co-localization of α-actinin-4 (grey) and ICAM-1 (false-colored, fire LUT) of a BMDC on an anti-MHCII+CD40L-coated surface (aMHCII+CD40L) after DMSO or 50μM blebbistatin 1h treatment. Representative of n=3 independent experiments. **E.** Representative TIRF images of CD40 after DMSO or 200μM Cytochalasin D (CytD) treatment on aMHCII+CD40L coated glass. **F**. Quantification of BMDCs size after CytD treatment (Unpaired Student's t-test, n_1=65 cells, n_2=44 cells, n_3=39 cells) **G.** Quantification of radial CD40 mean fluorescence intensity (MFI) after CytD treatment (Two-way ANOVA, n_1=78 cells, n_2=42 cells, n_3=33 cells). **H.** Representative TIRF images of CD40 after DMSO or 100μM CK666 1h treatment on aMHCII+CD40L coated glass. **I**. Quantification of BMDCs size after CK666 treatment (Unpaired Student's t-test, n_1=42 cells, n_2=68 cells, n_3=60 cells). **J**. Quantification of BMDCs solidity after CK666 treatment (Unpaired Student's t-test, n_1=42 cells, n_2=68 cells, n_3=60 cells) **K.** Quantification of radial CD40 mean fluorescence intensity (MFI) after CK666 treatment (Two-way ANOVA, n_1=50 cells, n_2=94 cells, n_3=83 cells). For all graphs, data are from three independent experiments (n=3); bars represent mean ± SD. *$p < 0.05$; ns, not significant.

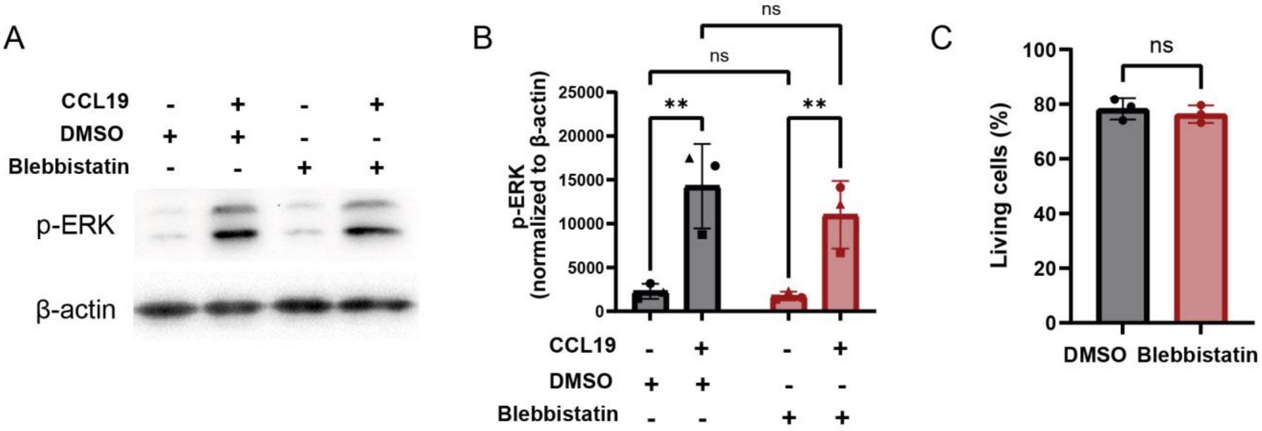

Supplementary Figure 7. Myosin inhibition does not affect CCL19-induced ERK activation or 24-hour BMDC survival. A. Representative western blots showing phosphorylation of ERK in BMDCs upon CCL19 activation, β-actin as loading control. LPS-matured BMDCs were treated with 50µM blebbistatin or DMSO (control) for 1 h at 37 °C before being stimulated for 2 min with 10nM of CCL19 when indicated. Cells were lysed and analyzed by western blot. **B**. Quantification of western blot data showing normalized levels of phosphorylated ERK to β-actin (One-way ANOVA, symbols represent data from the same independent experiment). **C.** LPS-matured BMDCs were treated with 50µM blebbistatin or DMSO (control) and stimulated with anti-CD40 monoclonal antibody (10 µg/mL) for 6 h at 37 °C. Cells were washed and cultured for 24h before being stained with Sytox Blue to measure cell viability by flow cytometry (Unpaired Student's t-test). For all graphs, data are from three independent experiments (n=3); bars represent mean ± SD. **p < 0.01; ns, not significant.
